# Multi-Omics Reveals the Inhibition of *Lactiplantibacillus plantarum* CCFM8724 in *Streptococcus mutans*-*Candida albicans* Mixed-Species Biofilms

**DOI:** 10.3390/microorganisms9112368

**Published:** 2021-11-16

**Authors:** Qiuxiang Zhang, Jiaxun Li, Wenwei Lu, Jianxin Zhao, Hao Zhang, Wei Chen

**Affiliations:** 1 State Key Laboratory of Food Science and Technology, Jiangnan University, Wuxi 214122, China; zhangqx@jiangnan.edu.cn (Q.Z.); 6190112052@stu.jiangnan.edu.cn (J.L.); luwewei@jiangnan.edu.cn (W.L.); jxzhao@jiangnan.edu.cn (J.Z.); zhanghao@jiangnan.edu.cn (H.Z.); 2 School of Food Science and Technology, Jiangnan University, Wuxi 214122, China; 3 (Yangzhou) Institute of Food Biotechnology, Jiangnan University, Yangzhou 225004, China; 4 National Engineering Research Center for Functional Food, Jiangnan University, Wuxi 214122, China

**Keywords:** *Streptococcus mutans*, *Candida albicans*, transcriptomics, metabolomics, biofilm, dental caries, *Lactiplantibacillus plantarum*

## Abstract

*Lactiplantibacillus plantarum* CCFM8724 is a probiotic with the potential to prevent dental caries in vitro and in vivo. To explore the effects of this probiotic at inhibiting *Streptococcus mutans*-*Candida albicans* mixed-species biofilm and preventing dental caries, multi-omics, including metabolomics and transcriptomics, was used to investigate the regulation of small-molecule metabolism during biofilm formation and the gene expression in the mixed-species biofilm. Metabolomic analysis revealed that some carbohydrates related to biofilm formation, such as sucrose, was detected at lower levels due to the treatment with the *L. plantarum* supernatant. Some sugar alcohols, such as xylitol and sorbitol, were detected at higher levels, which may have inhibited the growth of *S. mutans*. In transcriptomic analysis, the expression of the virulence genes of *C. albicans*, such as those that code agglutinin-like sequence (*Als*) proteins, was affected. In addition, metabolomics coupled with a Kyoto Encyclopedia of Genes and Genomes (KEGG) pathway analysis and RNA-seq revealed that the *L. plantarum* supernatant had an active role in sugar metabolism during the formation of the *S. mutans*-*C. albicans* mixed-species biofilm, and the *L. plantarum* supernatant was also related to carbohydrate utilization, glucan biosynthesis, and mycelium formation. Hence, *L. plantarum* CCFM8724 decreased the mixed-species biofilm mass from the perspective of gene expression and metabolic reprogramming. Our results provide a rationale for evaluating *L. plantarum* CCFM8724 as a potential oral probiotic for inhibiting cariogenic pathogen biofilm formation and improving dental caries.

## 1. Introduction

Dental caries, which is one of the most prevalent oral bacterial infectious diseases, represent a significant public health problem, not only in adults, but also in children [1]. *Streptococcus mutans* and *Candida albicans* have been detected in large amounts in oral plaque biofilms in children with early childhood caries (ECC), being considered to be directly related to ECC [2]. *S. mutans* is a key contributor to pathogenic dental biofilms, which can convert dietary sucrose into dextrans and acid [3]. *C. albicans* can robustly interact with *S. mutans* and has a significant impact on the virulence of dental plaque biofilms [4]. Common mechanical measures, such as tooth-brushing and mouth-washing, are effective in removing dental plaque biofilms; however, many children are not willing to brush their teeth. Probiotics and probiotic products, such as *Lactobacillus salivarius* [5] and *L. paracasei* NTU 101 fermented skim soy milk [6], can be used to remove biofilms in children. A major concern regarding the use of probiotics is the difficulty in maintaining their viability during shelf life. It seems that using the cell-free supernatants of probiotics can solve this problem.

At present, there is experimental evidence of the inhibition of dental plaque biofilms by *Lactobacillus* spp. *L. salivarius* inhibits *S. mutans* and *C. albicans* double-species caries biofilms by decreasing the amount of caries pathogenic species and reducing the biofilm mass in vitro [5]. Another study indicated that after taking a probiotic drink containing *L. casei* strain Shirota for 28 days, the abundance of *Veillonella* and *Kingella* increased significantly, while the abundance of some other bacteria related to caries was decreased in the oral cavity of young adults, as shown by the 16S rRNA sequencing results [7]. Different probiotic strains may prevent caries via different mechanisms, including the inhibition of cariogenic bacteria growth [8] and pathogenesis, exopolysaccharide (EPS) production reduction [9], quorum sensing inhibition [10], and reduction of the expression of key metabolic and virulence genes [11]. In our previous studies [12,13], we evaluated the anti-biofilm effects of *L. plantarum* CCFM8724 by reducing the dual-species biofilm mass and decreasing the caries score in dental caries rat models. However, how the *L. plantarum* CCFM8724 cell-free supernatant affects this cariogenic *S. mutans* and *C. albicans* mixed-species biofilm environment and how it affects the biofilm metabolome and transcriptome remains unclear.

To explore the mechanism of extracellular probiotic metabolites of *L. plantarum* CCFM8724 inhibiting dual-species biofilms, we employed a combination strategy using untargeted metabolomics and transcriptomics, revealing the distinct metabolism and gene expression characteristics that are important for *S. mutans* and *C. albicans* mixed-species biofilm formation. This work reveals the mechanism of microbe–microbe interaction and provides promising baseline information for the potential use of this probiotic.

## 2. Materials and Methods

### 2.1. Chemicals and Materials

Methanol (high-performance liquid chromatography (HPLC) grade) and acetonitrile (HPLC grade) used for metabolite extraction were purchased from Merck KGaA (Darmstadt, Germany). Pyridine, N-methyl-N-(tri-methylsilyl) trifluoroacetamide with 1% trimethylchlorosilane (MSTFA + 1% TMCS) and methoxyamine hydrochloride (MeOX) used for metabolite derivatization were obtained from Sigma-Aldrich (St. Louis, MO, USA).

### 2.2. Bacterial Strains and Growth Conditions

*L. plantarum* CCFM8724 was cultured in MRS broth (Difco^TM^, Detroit, MI, USA) under anaerobic conditions at 37 °C for 24 h. The supernatant was collected by centrifugation at 6000× *g* at 4 °C for 10 min and filtered through a 0.22 μm sterile membrane, then stored at 4 °C before use. *S. mutans* ATCC 25175 and *C. albicans* ATCC18804 were purchased from the China General Microbiological Culture Collection Center (CGMCC, Beijing, China). *S. mutans* was inoculated in tryptic soy broth (TSB, DifcoTM, Detroit, MI, USA) and *C. albicans* was cultured by yeast extract peptone dextrose medium (YPD, DifcoTM, Detroit, MI, USA) under aerobic conditions at 37 °C. When generating the dual-species biofilm, 5% sucrose was added to the medium.

### 2.3. Sample Preparation for Metabolomics and Transcriptomics Analysis

For each replicate, 75 μL of *S. mutans* culture suspension and equal *C. albicans* culture suspension were added into each well of a 96-well microtiter plate. The number of *S. mutans* and *C. albicans* per well was 10^7^ and 10^6^ CFU mL^−1^, respectively. A total of 50 μL of *Lactobacillus* supernatant was added at the same time. The same volume of MRS broth instead of supernatant was used as control. Biofilms were formed in a 96-well microtiter plate for 24 h at 37 °C. Then, the contents of each well were removed and washed twice with phosphate buffer saline (PBS) to remove the planktonic cells. Next, the biofilm was scraped by using 200 μL non-enzyme pipette tips and finally pooled together to collect approximately 50 mg of biofilm mass for metabolite extraction and RNA-seq analysis [14]. After quick freezing in liquid nitrogen, the frozen cells were stored at −80 °C for further use. Metabolomics samples from each group had 6 replicates. Transcriptomics samples from each group had 2 replicates.

### 2.4. Untargeted Metabolomics by Gas Chromatography–Mass Spectrometry

The frozen sample was added with 0.4 mL ice-cold extraction solvent (acetonitrile:methanol:water) = 2:2:1(*v*/*v*/*v*). A total of 20 μL of ethyl undecanoate (C21:0) (0.3 μmol/sample) and 100 μL of magnetic beads were added into each sample before extraction. The C21:0 was used as an internal standard. The mixture was homogenized in a ball mill for 45 sec at 65 Hz and stopped for 15 s [15]. The homogenisation cycle was performed ten times. The mixture was then centrifuged at 12,000× *g* for 15 min at 4 °C. The supernatant of the extracts was dried in a vacuum centrifuge (RC1022, Thermos, Waltham, MA, USA) and resuspended in MeOX-pyridine and MSTFA with 1% TMCS for derivatization. For each group, six parallel experiments were completed [16].

GC–MS (Gas chromatography–mass spectrometry) analysis was performed on a Thermo trace 1310 gas chromatograph coupled to an Thermo TSQ8000_evo quadrupole mass selective detector equipped with an RTX-5MS capillary column (0.25 mm diameter, 0.25 μm film thickness). The detection and metabolite identification using metabolomics were performed as the methods described by Lu [16]. Briefly, the derivatized extract (1 μL) was injected into the GC–MS system in split mode (split ratio 10). The initial GC oven temperature rose from 50 °C to 230 °C at 5 °C/min, then to 320 °C at a rate of 90 °C/min and held for 5 min. The MS was operated in a scan range of 33 to 600 *m*/*z*. The transfer line and ion source temperatures were 280 °C and 300 °C, respectively. The electron impact mode was operated at 70 eV.

The obtained data of GC–MS were analysed as followed. ABF converter 4.0 was used to convert “raw” format files into “abf” format. The MSDIAL3.48 equipped with the FiehnLib database was used for raw peaks exaction, retention time adjustment, peak alignment, deconvolution analysis, and peak identification as reported in a previous study [17]. Statistical analysis and pathway enrichment analysis were performed using MetaboAnalyst 5.0 online software (https://www.metaboanalyst.ca/, accessed on 13 October 2021).

### 2.5. RNA Extraction and Illumine Sequencing

Total RNAs were isolated from frozen biofilms using TRIzol (Thermo Fisher Scientific, Shanghai, China) and the DNA-free kit (Ambion (Thermo Fisher Scientific), Shanghai, China). Purity and concentration of RNAs were checked using the NanoPhotometer^®^ spectrophotometer (IMPLEN, Westlake Village, CA, USA), and Qubit^®^ RNA assay kit in Qubit^®^ 2.0 Flurometer (Life Technologies, Carlsbad, CA, USA). Library preparation for strand-specific transcriptome sequencing and RNA-seq were performed by Novogene (Tianjin, China). Briefly, the library of each sample was generated by 1 μg of RNA and then sequenced using the Illumina Hiseq platform (125 bp/150 bp paired-end reads). Raw data were processed by custom Perl scripts to obtain the clean data. The clean reads were mapped to the *S. mutans* reference genome (https://www.ncbi.nlm.nih.gov/genome/?term=ATCC+25175 accessed on 13 October 2021) and *C. albicans* reference genome (accessed on 13 October 2021, https://www.ncbi.nlm.nih.gov/genome/?term=Candida+albicans) using the Hisat2 software program with default parameters. On the basis of the clean data, the edgeR package was adopted to identify differentially expressed genes (DEGs) using the following criteria: adjusted *p* value < 0.05 and log2FC > 1. DEGs were subjected to a Kyoto Encyclopedia of Genes and Genomes (KEGG) pathway analysis [18]. Each group had 2 replicates.

### 2.6. DNA Extraction and RT-qPCR

DNA was extracted from the biofilm according to the protocol in FastDNA SPIN Kit for Soil 50T (MP Biomedicals, California, USA). Quantitative real-time PCR (qPCR) was performed according to the protocol provided by iTaq™ Universal SYBR^®^Green Supermix (Bio-Rad Laboratories, Inc., Shanghai, China), in a CFX Connect^TM^ real-time system (Bio-Rad Laboratories, Inc.). From the real-time PCR data, Cq values were obtained to calculate each organism abundances. RT-qPCR primers are listed in Table S1.

### 2.7. Comparative Genomics and Enzyme Analysis

The OrthoMCL software program was used for the homologous gene analysis. Open reading frames in the genome were predicted to obtain the amino acid sequence of enzymes based on the Clusters of Orthologous Groups of protein (COGs) and KEGG databases. The HMMER3.1 software program was used to predict from amino acid sequences aligned to the Carbohydrate active enzymes database (CAZy).

### 2.8. MultiStatistical Analysis and Visualization

R studio 4.0.1 with the R-packages ggplot2 and ggsci was used to visualize the results. The differences in this research between groups were determined by a *t*-test. In the metabonomic analysis, a *p* value < 0.05 indicated statistical significance. In pathway enrichment, a false discovery rate (FDR) analysis was performed to adjust the *p* value to *q* value. A *q* value < 0.05 indicated statistical significance.

## 3. Results

### 3.1. Analysis of Untargeted Metabolomic PCA and PLS-DA Data

**Figure 1:**
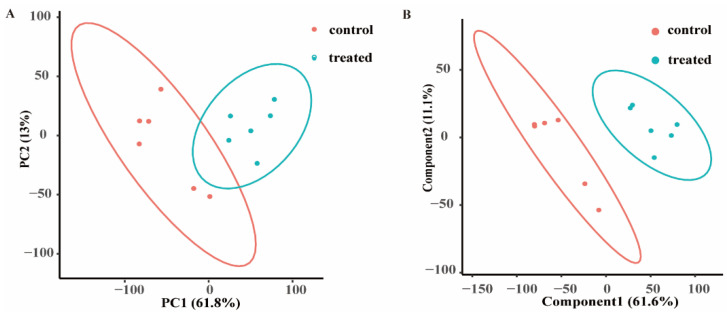
The metabolites in treated group compared to those in control group. (**A**) Principal component analysis (PCA) score plot of metabolite profiles from the treated and control groups. (**B**) Partial least squares discriminant analysis (PLS-DA) score plot of metabolite profiles from the treated and control groups. Each point represents an independent biological replicate. The red dots indicate the control group, the blue dots indicate the treated group.

Untargeted metabolomics is an omics method in systems biology, via which the changes in all small-molecule metabolites between groups, which can reflect the different biological processes in cells, are analysed [14]. Metabolites with high-quality peak signals were collected to obtain reliable metabolome data (Table S2). Mixed-species biofilms were treated with *L. plantarum* supernatant or MRS broth and were subjected to untargeted metabolomics to determine the effect of small molecules in *L. plantarum* in the biofilm formation. We first used principal component analysis (PCA) to visualize the metabolomic similarities of the samples in a group and the differences in samples between groups. PCA can project complex GC–MS data into a lower dimensional space, and the biofilm samples marked with different colours were projected onto a two-dimensional space (principal components 1 and 2 (PC1 and PC2)), and clustered separately. PC1 and PC2 were responsible for 61.8% and 13% of the variation, respectively, indicating that the two groups could be distinguished based on their metabolic compounds (Figure 1A).

Similar to PCA, partial least squares discriminant analysis (PLS-DA) is a supervised extension of PCA that can clearly separate different groups of data [14]. The score plot (Figure 1B) that resulted from the PLS-DA overview of the biofilm metabolome data revealed that intracellular small-molecule metabolism was significantly changed by the treatment with *L. plantarum*.

### 3.2. Differential Metabolites via Biofilm Profiling Analysis

**Figure 2:**
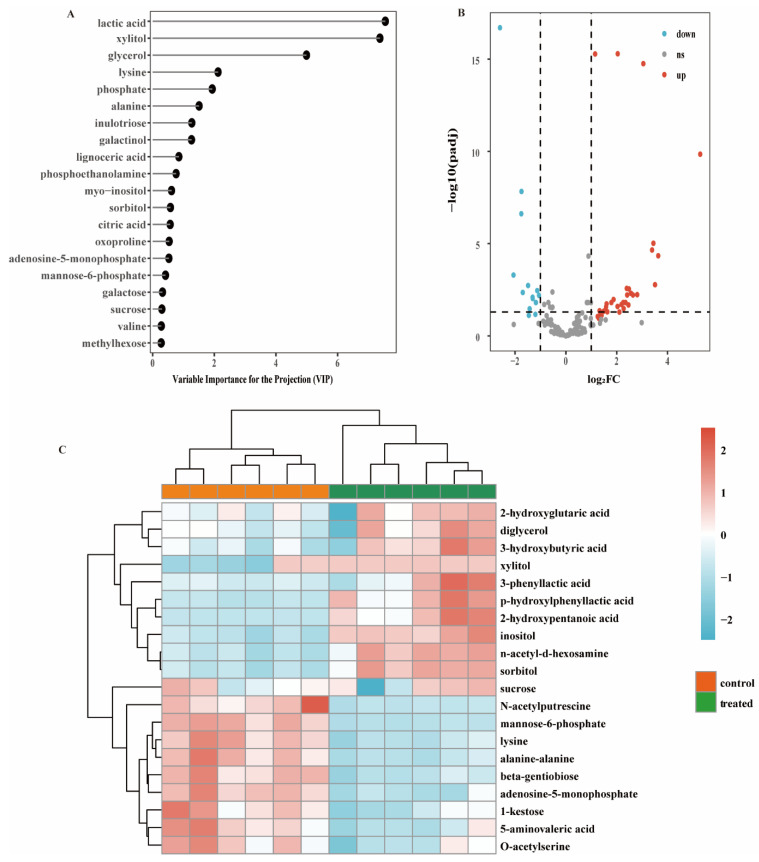
Significantly changed metabolites between groups. (**A**) Variable importance for the projection (VIP) score calculated by PLS-DA. (**B**) Volcano plot of the metabolites from the treated and control biofilms. The blue dots indicate the downregulated metabolites, the red dots indicate the upregulated metabolites, and the grey dots indicate the non-differentially expressed metabolites. (**C**) Heatmap of top 20 significantly changed metabolites. Cluster 1 and cluster 2 represent the two clusters of control and treated group, respectively. The six columns for each cluster represent independent metabolomics replicates.

Collectively, the data analysis of the biofilm metabolites yielded 216 metabolites. In the PLS-DA models, metabolites with a variable importance for the projection (VIP) value greater than 1 made a significant contribution to the separation between the control and treatment groups [19]. The VIP plots (Figure 2A) demonstrated that some of the identified metabolites contributed to class separation.

The DEseq statistical method was used to identify the significant differences between groups. Overall, 45 metabolites were identified (*p* < 0.05, log_2_(fold change (FC)) > 1). As shown in Figure 2B, 14 metabolites were downregulated (blue dots, *p* < 0.05, log_2_FC ≤ −1), 31 metabolites were upregulated (red dots, *p* < 0.05, log_2_FC ≥ 1), and the others were non-differentially expressed (grey dots, *p* > 0.05 or −1 < log_2_FC < 1). To display the differences in the metabolite content and the similarities in mixed-species biofilms, only the significantly changed metabolites (top 20) were selected to construct a heatmap (Figure 2C). Some sugars, such as sucrose, mannose-6-phosphate, and 1-kestose are potential metabolites resulting from the formation of *S. mutans*-*C. albicans* mixed-species biofilm. Some organic acids, such as 3-phenyllactic acid and p-hydroxylphenyllactic acid, were upregulated.

### 3.3. Metabolic Pathways

**Figure 3:**
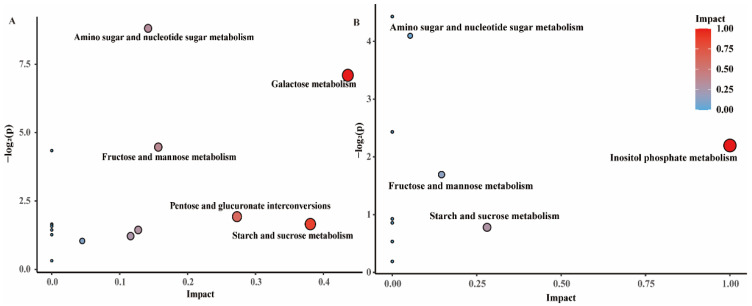
Pathway enrichment analysis (PEA) and pathway topology analysis (PTA) results. (**A**) Pathway enriched in *C. albicans*. (**B**) Pathway enriched in *S. mutans*.

To further explore the detailed metabolic pathways in which a dual-species biofilm is affected by the *L. plantarum* supernatant, a KEGG pathway impact analysis was performed based on the differential metabolites. The KEGG results, including the pathway enrichment analysis (PEA) and pathway topology analysis (PTA) results, were analysed (Tables S3 and S4). The PTA results from the KEGG are shown on the *x*-axis. The larger the circle size, the higher the centrality of the metabolite involved in the corresponding pathway is. The enrichment analysis (PEA) KEGG results are presented on the *y*-axis. The closer the circle gets to red, the more significant the change in the compound is [20]. Five major KEGG pathways significantly impacted were detected in *C. albicans*, which were amino sugar and nucleotide sugar metabolism, galactose metabolism, fructose and mannose metabolism, pentose and glucuronate interconversions, and starch and sucrose metabolism, as shown in Figure 3A. In addition, four major metabolic pathways were affected in *S. mutans*: inositol phosphate metabolism, fructose and mannose metabolism, starch and sucrose metabolism, and amino sugar and nucleotide sugar metabolism (Figure 3B).

### 3.4. Global Changes at the Transcriptome Level

To investigate how the *L. plantarum* supernatant affects the cell metabolism of *S. mutans* and *C. albicans*, the mRNA profiles of the treatment and control groups were analysed using RNA-seq. A total of 32.98 Gb of data were acquired, including 223,211,718 raw reads and 219,913,474 clean reads. The error rate of single-base location sequencing in all four samples was 0.03%. The Q20 and Q30 percentages were higher than 97% and 93%, respectively, indicating the high quality of the data. However, *S. mutans* did not reach a high sequencing depth (Table 1), probably because of the inhibition of probiotics. Therefore, only the transcriptome data of *C. albicans* were analysed.

To verify the reliability of the RNA-seq data, absolute quantitative RT-PCR was applied to detect the abundance of *S. mutans* and *C. albicans* in mixed-species biofilms in the treatment and control groups. Standard curves were obtained for *S. mutans* and *C. albicans* and are shown in Figure S1A,B. The number of colonies of *S. mutans* treated with the *L. plantarum* supernatant was reduced by more than 3 logs, while the number of *C. albicans* colonies was in a similar range compared with that of the control (Table 2), and this result was similar to the results obtained using RNA-seq.

### 3.5. Differentially Expressed Genes (DEGs) between the Biofilms of the Treated and Control Groups

**Figure 4:**
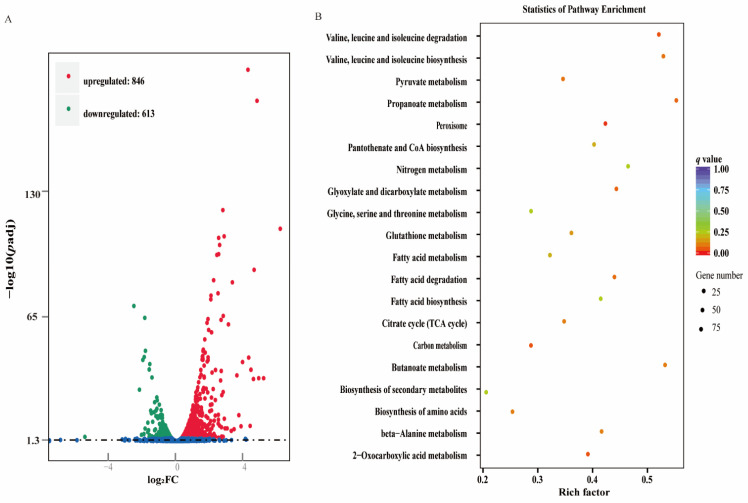
(**A**) Volcano map of differentially expressed genes (DEGs) for treated vs. control. Different colours (red, green, and blue) represent upregulated, downregulated, and no significant changes, respectively. (**B**) The most abundant KEGG pathways for differentially expressed genes.

RNA sequencing technology was used to reveal the inhibitory mechanism of the *L. plantarum* supernatant on the biofilms consisting of *S. mutans* and *C. albicans*. The volcanic maps show the overall distribution of the differentially expressed genes between the two groups. A total of 1459 important DEGs, 613 downregulated genes, and 846 upregulated genes were identified in the experimental group (Figure 4A and Table S5). Next, we performed KEGG signalling pathway enrichment analyses. Compared with the control group, 1459 DEGs were enriched in 97 pathways in *C. albicans* after treatment with the *L. plantarum* supernatant. The 20 most abundant pathways are shown in Figure 4B. The size of the dots represents the number of genes. The closer the *q* value is to 0, the greater the extent of enrichment.

**Figure 5:**
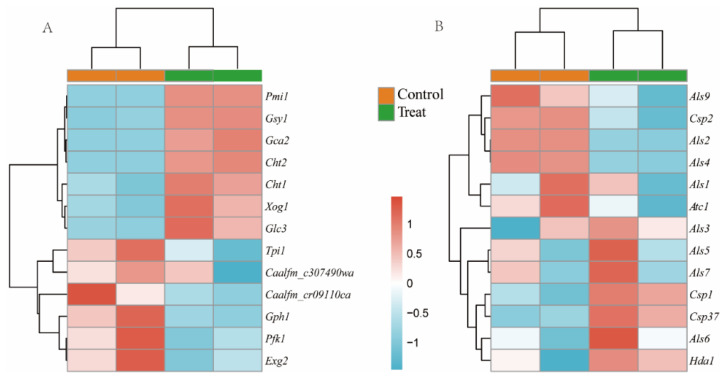
Heatmap for gene expression in *C. albicans*. (**A**) Genes related to carbohydrate metabolism. (**B**) Genes related to hyphae formation.

As shown in Figure 3A, the carbohydrate metabolism pathways, including starch and sucrose metabolism, galactose metabolism, amino sugar and nucleotide sugar metabolism, and fructose and mannose metabolism, were related to some genes. Therefore, we rearranged the 13 DEGs of the carbohydrate metabolism pathway in *C. albicans* (Figure 5A). The expression of some genes related to carbohydrate metabolism changed significantly with the addition of *L. plantarum*. In addition, the gene expression of hyphae in *C. albicans* is closely related to virulence [21]. Therefore, we rearranged another 13 DEGs related with hyphae formation in Figure 5B. The expression of genes associated with the filamentous growth of fungi, such as some genes of the *Als* gene family, was downregulated.

### 3.6. Integrated Metabolome and Transcriptome Analysis

**Figure 6:**
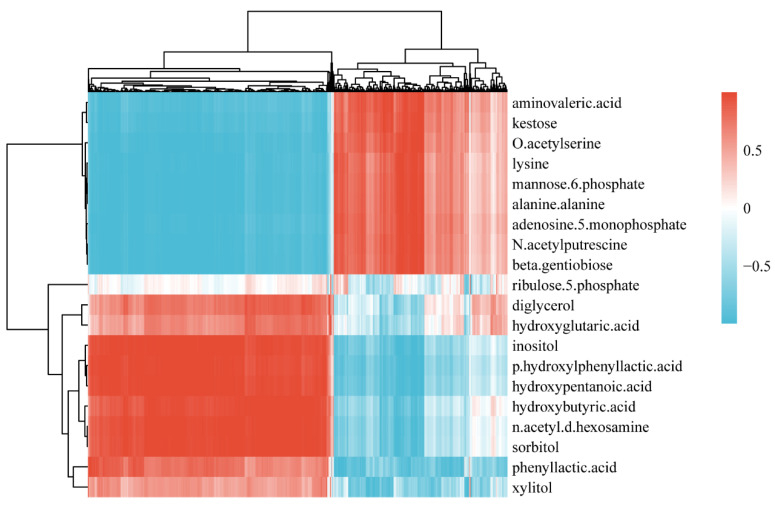
The heatmap of the correlation between differentially expressed genes (DEGs) and metabolites in *C. albicans*.

To clearly reveal the bacterial–fungal interactions in the dual-species biofilm, we combined transcriptomics and metabolomics. We performed a Pearson’s correlation coefficient analysis based on the metabolome and transcriptome profiles. Based on a Pearson correlation coefficient > 0.9, the screening results showed that some metabolites were significantly correlated with the DEGs, as shown in Figure 6. The important DEGs related to the metabolites of *C. albicans* in the mixed-species biofilm are listed in Table 3. Most of the DEGs related to sugar metabolites in *C. albicans* showed a negative correlation, while most of the DEGs related to sugar alcohol and organic acid showed a positive correlation. Mannose-6-phosphate and kestose were related to 131 and 158 DEGs, respectively.

### 3.7. Genomics Analysis of L. plantarum CCFM8724

**Figure 7:**
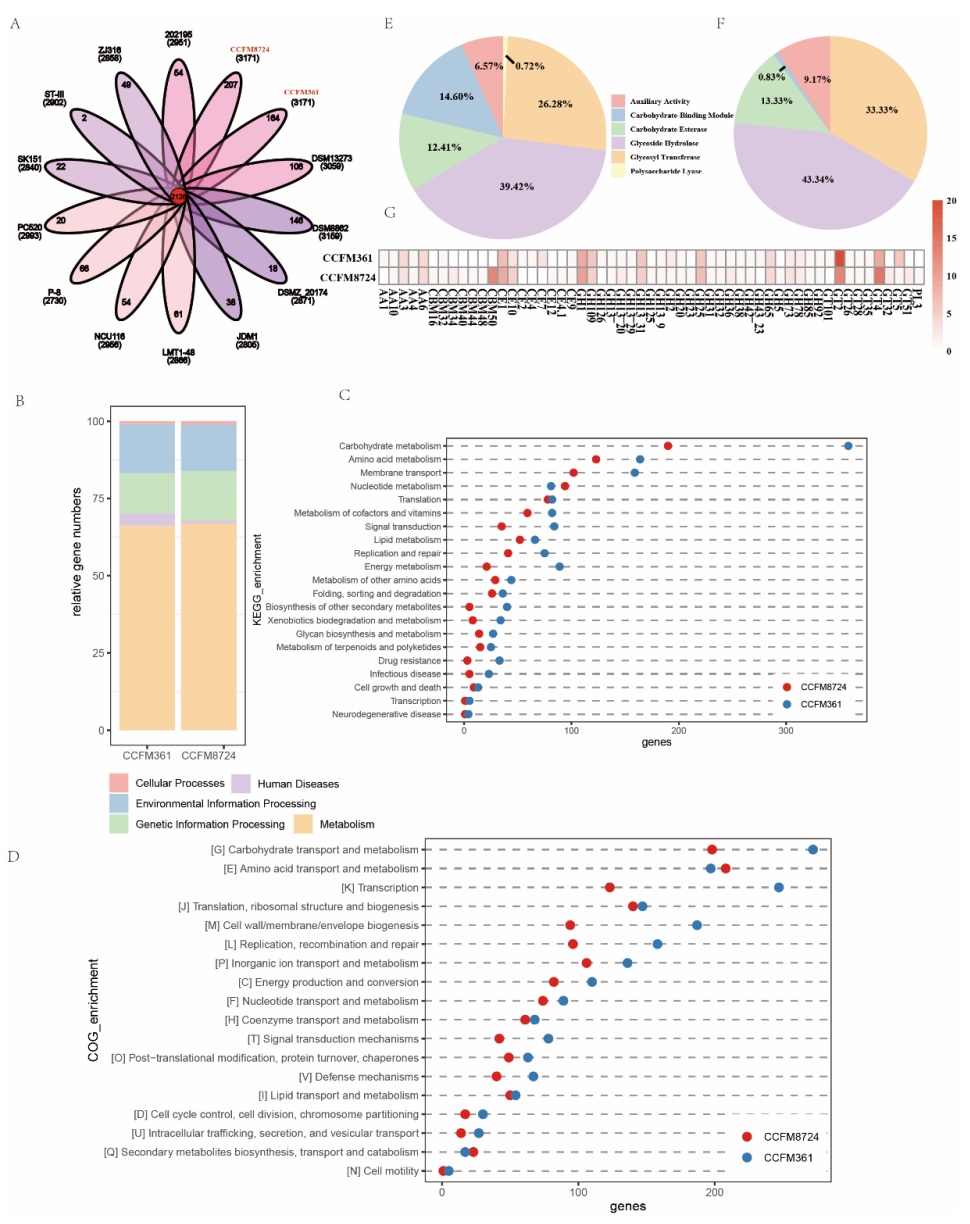
Comparative genome analysis of *L. plantarum* CCFM8724 and CCFM361. (**A**) Venn plot of shared and unique core genes distribution among *L. plantarum* strains. (**B**) The level I of Kyoto Encyclopedia of Genes and Genomes (KEGG) pathway enrichment analysis. (**C**) The level II of KEGG pathway enrichment analysis. (**D**) Enrichment analysis of Clusters of Orthologous Groups of protein (COGs) functional categories. (**E**) Genome-wide comparative distribution of carbohydrate-active enzymes (CAZy) in *L. plantarum* CCFM8724. (**F**) Genome-wide comparative distribution of CAZy in *L. plantarum* CCFM361. (**G**) Identification and quantification of gene count coding for different CAZy families of two strains. AA, auxiliary activity; CBM, carbohydrate-binding module; CE, carbohydrate esterase; GH, glycoside hydrolase; GT, glycosyltransferase; PL, polysaccharides.

To reveal at the molecular level why *L. plantarum* CCFM8724 can inhibit double-species biofilm and change the metabolic and transcriptomic level, we performed a genomics analysis of *L. plantarum* CCFM8724, taking *L. plantarum* CCFM361 as the control strain, from the same subspecies, without the effect on inhibiting double-species biofilm [13]. Through a homologous gene analysis with the reported complete *Lactobacillus* genome, *L. plantarum* CCFM8724 and CCFM361 shared 2126 core genes. *L. plantarum* CCFM8724 had 207 unique core genes, and CCFM361 had 164 unique core genes (Figure 7A). The gene function was annotated using the COG and KEGG databases (Figure 7B–D). Genes related to carbohydrate metabolism showed the greatest count difference between the two strains (Figure 7B–D). Thus, enzymes involved in carbohydrate utilization were further analysed. Putative protein-coding sequences were predicted and annotated using the CAZy database. *L. plantarum* CCFM8724 had more genes encoding carbohydrate binding modules (CBMs) and polysaccharide lyases (PLs), but fewer genes encoding carbohydrate esterases (CEs), glycoside hydrolases (GHs), auxiliary activity (AA), and glycosyltransferases (GTs) (Figure 7E,F). The number of genes encoding the enzymes involved in carbohydrate utilization is shown in Figure 7G.

## 4. Discussion

In our previous studies [12,13], the inhibition of *S. mutans-C. albicans* mixed-species biofilms was observed when the *L. plantarum* supernatant was added. In this study, we employed multi-omics to reveal the distinct metabolic reprogramming and gene expression during *S. mutans*-*C. albicans* dual-species biofilm formation with the addition of the *L.* *plantarum* supernatant.

Metabolic data revealed that most of the impacted metabolites were involved in the carbohydrate metabolism (Figure 2A). Sucrose, a positively changed metabolite, was shown to be the most cariogenic of all carbohydrates [22]. After *L. plantarum* supernatant treatment, the intracellular remaining sucrose increased. Sucrose can be utilized to generate extracellular polymeric substances (EPS) to help cells form biofilms [23]. A diet rich in sucrose enhances the levels of *S. mutans* in dental biofilms, especially in the presence of *C. albicans* in a caries rat model [24]. Sucrose degradation products can also be easily used by *C. albicans*, thus promoting its growth [25]. Importantly, *S. mutans* has multiple pathways that use sucrose, such as cutting the α-1,2-linked bond of sucrose to generate organic acids and converting sucrose into extracellular polymer glucan via several glycosyltransferase enzymes (Gtfs) [26]. The biofilm is partly composed of extracellular polymer glucan. The mannose-related compound phosphorylated mannose-6-phosphate was detected at a higher level in the control group (Figure 2C). Mannose-6-phosphate was related to 131 DEGs (Table 3). Similar to sucrose, mannose is also associated with EPS formation, and one of the components of EPS, called Pel, governs biofilm formation and is composed of mannose [27].

In contrast, the xylitol and sorbitol levels were higher in the treatment group than in the control group (Figure 2C). Burt et al. [28] reported that xylitol-sweetened gum was not only noncariogenic, but also had an anticariogenic effect. Chi [29] also found that xylitol-sweetened milk significantly decreased the *S. mutans* abundance compared to sucrose-sweetened milk. Moreover, it is clear that sorbitol can reduce the acid production of *S. mutans* in vitro, inhibit acid production in dental plaque in vivo, and prevent dental caries [30]. These results also indicate that the xylitol and sorbitol produced by *L. plantarum* may be the antimicrobial material basis, which requires further studies.

The mixed-species biofilms cultured in MRS broth were able to generate organic acids, such as 5-aminovaleric acid (Figure 2C). The organic acids produced by *S. mutans* can quickly lower the environmental pH, while *S. mutans* is also tolerant to low pH [31]. Cornejo et al. [32] performed population genetic analyses on the core genome of *S. mutans* and identified 73 unique core genes, most of which are related to carbohydrate metabolism and acid resistance. However, some organic acids, such as 3-phenyllactic acid and p-hydroxylphenyllactic acid, were detected at higher levels in the treated group (Figure 2C). In addition, lactic acid had the highest contribution for distinguishing the control and treatment groups (Figure 2A). It has been reported that the organic acids and fatty acids produced by *Lactobacillus*, such as lactic acid, acetic acid, and capric acid, have good bacteriostatic effects on *Escherichia coli* [33]. We found that 3-phenyllactic acid and p-hydroxylphenyllactic acid can be generated from *L. plantarum* [34]; therefore, the organic acids produced by *L. plantarum* may inhibit the growth of *S. mutans*. Coincidently, the reads corresponding to *S. mutans* were not detected in the RNA-seq analysis, indicating that *S. mutans* was inhibited in the mixed-species biofilm after the treatment with the *L. plantarum* supernatant. To confirm this inhibition, absolute quantitative RT-PCR was used to detect the number of colonies of *S. mutans* and *C. albicans* in the mixed-species biofilm (Table 3). The number of *C. albicans* colonies was in a similar range as that of the control group, while the number of *S. mutans* colonies was decreased significantly, and this result was similar to the RNA-seq results.

The combination of transcriptomics and metabolomics can clearly reveal the bacterial–fungal interactions in mixed-species biofilms. The DEGs in the treatment and control groups resulting from the transcriptomic analysis of *C. albicans* are shown in Figure 4A. A large number of unigenes in *C. albicans* were classified into various KEGG metabolic pathways (Figure 4B). The KEGG pathways “TCA cycle”, “carbon metabolism,” and “pyruvate metabolism” were significantly enriched, compared to untargeted metabolomics. As shown in Figure 5A, we found 13 DEGs of the carbohydrate metabolism pathway. Among these genes, *Pfk1* participated in both fructose and mannose metabolism and galactose metabolism, and its expression levels were significantly reduced compared to those in the control group. Meanwhile, some of the gene expression levels were increased, suggesting that *C. albicans* requires a more vigorous carbohydrate metabolism to meet its growth needs without the crossbreeding with *S. mutans* [25]. In addition, the genes *Exg2* and *Tpl1* were not only associated with the carbohydrate metabolism pathway, but also with the cell wall components, such as mannosan and glucan, which were downregulated.

Contrarily, we observed that the genes related to the filamentous growth of fungi, including *Als1*, *Als2*, *Als4*, and *Als9*, were downregulated, as expected. Our findings were in agreement with those of James et al., who reported a similar gene expression trend in *C. albicans* under the treatment with a combination of *L. plantarum*, *L. helveticus*, and *Streptococcus salivariuin* [35]. A family of cell surface proteins called agglutinin-like sequence (*Als*) proteins is associated with cell adhesion and biofilm formation [36]. The results showed that the *L. plantarum* supernatant inhibited the adhesion ability of *C. albicans.* Furthermore, the genes associated with the cell wall components (*Csp2* and *Atc1*) were downregulated.

Apart from the genes mentioned above, other important genes related to the metabolites of *C. albicans* in mixed-species biofilms are listed in Figure 6 and Table 3. Adenosine-5-monophosphate was detected at a low level after the treatment with the *L. plantarum* supernatant and was related to 140 DEGs. Adenosine-5-monophosphate, an organic compound consisting of adenine, ribose, and phosphoric acid, is a component of the extracellular DNA (eDNA). eDNA is an important biofilm component that was recently discovered. A study by Lucio [37] indicated that eDNA can be found in some species present in biofilms, such as *Pseudomonas aeruginosa*, *S. intermedius*, and *S. mutans*.

Finally, we demonstrated that the ability of carbohydrate utilization is the major genomic difference between two *L. plantarum* strains (*L. plantarum* CCFM8724 and CCFM361) in Figure 7B–D. This helps to explain why intracellular carbohydrate metabolism of dual-species biofilm (Figure 2) in the treated group is the most variable. As shown in Figure 7E, F, the *GH* family, the highest proportion of the two strains, is a critical enzyme group accounting for the bacterial adaptation capacity to the host’s environment, through hydrolysing the dietary and host-produced carbohydrates [38]. In addition, *L. plantarum* CCFM8724 has a higher *CBM* level, which can combine with *GH* family to degrade chitin or peptidoglycan to inhibit the pathogen growth, especially the *CBM50* (Figure 7G). It can be inferred from the results of the genomic analysis that *L. plantarum* CCFM8724 had unique carbohydrate utilization ability to yield some bioactive compounds, and in turn, to yield its beneficial effect in the oral environment through either inhibiting the biofilm formation or interacting with oral microbes. However, we also acknowledge the limitation that the specific compounds remain unclear, which deserves further study.

## 5. Conclusions

In conclusion, the multi-omics analyses provide new insights, showing the inhibition of the *L. plantarum* CCFM 8724 supernatant; in the metabolomics analysis, because the carbohydrate metabolism was deeply influenced, the crossbreeding of *C. albicans* and *S. mutans* was changed. In the transcriptomic analysis, the expression of virulence genes, such as those that code *Als* (agglutinin-like sequence) proteins, was affected. Our results strongly confirm that *L*. *plantarum* CCFM8724 can decrease the biofilm mass, regardless of the gene expression or metabolic reprogramming; therefore, it has the potential to act as a therapeutic agent for the prevention and treatment of caries.

## Figures and Tables

**Table 1:** RNA-seq in mixed-species biofilms.

Sample Name	C1	C2	T1	T2
Total reads (*C. albicans*)	5,5819,264	54,481,928	56,579,976	53,032,306
Total mapped (*C. albicans*)	36,144,061 (64.75%)	35,817,034 (65.74%)	48,178,839 (85.15%)	44,747,300 (84.38%)
Total reads (*S. mutans*)	55,506,496	54,041,988	56,174,376	52,584,766
Total mapped (*S. mutans*)	13,877,006 (25%)	12,460,931 (23.06%)	18,215 (0.03%)	45,311 (0.08%)

**Table 2:** The number of organisms detected by quantitative real-time PCR (qPCR).

Organisms	Cq		Cells	
	C	T	C	T
*S. mutans*	15.18 ^a^	24.36 ^b^	10^9 a^	10^6 b^
*C. albicans*	16.15 ^a^	16.22 ^a^	10^10 a^	10^10 a^

Note: The equations of linear regressions of DNA copies vs. cycle quantification are characterized by their slope, *y*-axis intersection and the R^2^ values in Figures S1 and S2. Cells numbers were theoretical values based on the copies of DNA. ^a^ and ^b^ represent the difference in the cells in each row.

**Table 3:** Some representative metabolites and significantly correlated differentially expressed genes (DEGs) of *C. albicans* between groups.

Compounds	Correlated DEGs
Mannose-6-phosphate	131
Kestose	158
Lysine	127
Adenosine-5-monophosphate	140

## Data Availability

Data used to support the findings of this study are available from the corresponding author upon request.

## Supplementary Materials

The following are available online at https://www.mdpi.com/article/10.3390/microorganisms9112368/s1, Figure S1: Standard curve of *S. mutans* by RT-Q-PCR, Figure S2: Standard curve of *C. albicans* by RT-qPCR, Table S1: Oligonucleotide primer pairs used for qPCR, Table S2: Differentially expressed metabolites between treat group and control group. Table S3: KEGG pathway impact analysis of differentially expressed metabolites in *S. mutans*. Table S4: KEGG pathway impact analysis of differentially expressed metabolites in *C. albicans*. Table S5: Differentially expressed genes of *C. albicans* between control group and treated group.
