# Supplementary material for: Multi-Omics Reveals the Inhibition of Lactiplantibacillus plantarum CCFM8724 in Streptococcus mutans-Candida albicans Mixed-Species Biofilms
Source: Microorganisms. 2021 Nov 16;9(11):2368. doi: 10.3390/microorganisms9112368 (PMC8619341; doi:10.3390/microorganisms9112368)
Supplement: Supplementary file 1 [file microorganisms-09-02368-s001.zip › microorganisms-1459600-supplementary.pdf]

Supplementary Materials

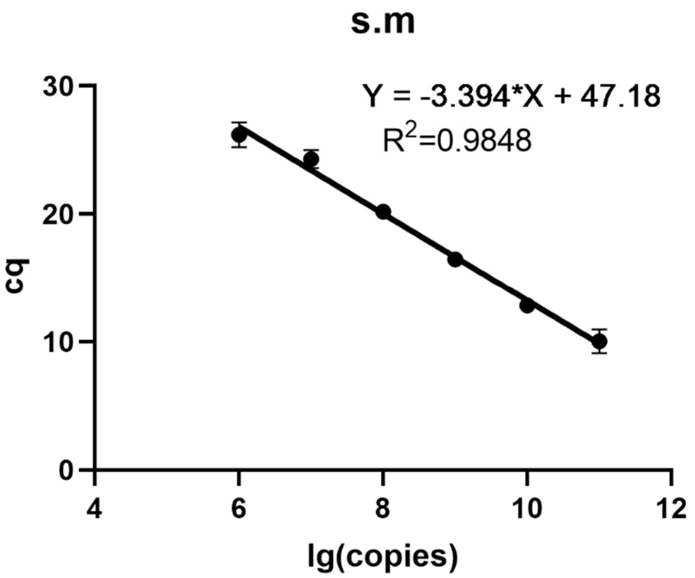

Figure S1. Standard curve of *S. mutans* by RT-Q-PCR

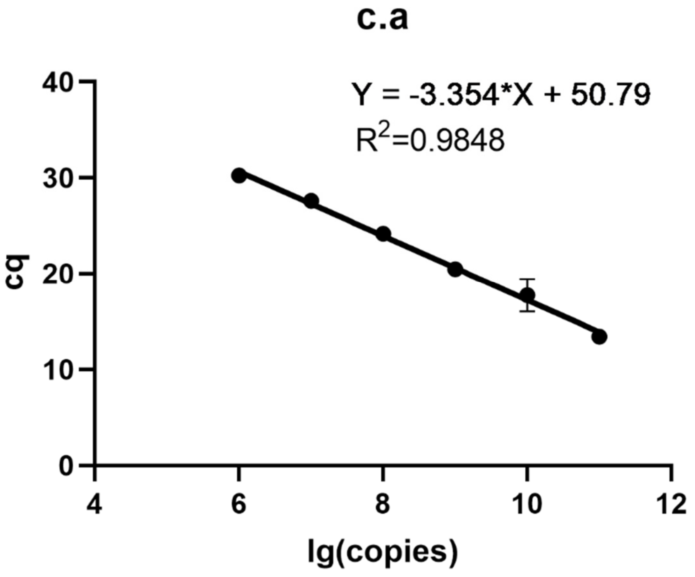

Figure S2. Standard curve of *C. albicans* by RT-Q-PCR

Table S1. Oligonucleotide primer pairs used for qPCR

Genes		Primer sequences
S. mutans	*S. mutans*-F	GATAATTGATTGAAAGATGCAAGC

	S. mutans-R	ATTCCCTACTGCTGCCTCCC
C.a	*C. albicans*-F	TTTATCAACTTGTCACACCAGA
	C. albicans-R	ATCCCGCCTTACCACTACCG

Table S2. Differentially metabolite between treat group and control group

Metabolite	Log2FC	P value	Padj.
mannose-6-phosphate	-3.63095453	6.59E-67	9.42E-65
N-acetylputrescine	-2.593982916	2.76E-19	1.97E-17
3-phenyllactic acid	2.039795785	1.07E-17	5.08E-16
diglycerol	1.15040773	1.46E-17	5.21E-16
p-hydroxylphenyllactic acid	3.046934749	6.11E-17	1.75E-15
2-hydroxypentanoic acid	5.286609398	5.90E-12	1.41E-10
beta-gentiobiose	-1.747533765	7.25E-10	1.48E-08
lysine	-1.754605151	1.34E-08	2.39E-07
n-acetyl-d-hexosamine	3.443896165	5.97E-07	9.49E-06
sorbitol	3.391851871	1.54E-06	2.20E-05
inositol	3.634116726	3.48E-06	4.52E-05
2-hydroxyglutaric acid	0.886450542	3.96E-06	4.71E-05
adenosine-5-monophosphate	-2.061602146	4.57E-05	0.000502379
xylitol	3.508007941	0.000164197	0.001677151
5-aminovaleric acid	-1.487088627	0.000194563	0.001854838
3-hydroxybutyric acid	2.394861353	0.00029757	0.002659535
ribulose-5-phosphate	2.478947262	0.000336086	0.002827079
1-kestose	-1.128613708	0.000438451	0.003483247
O-acetylserine	-0.53017041	0.00055217	0.004155804
alanine-alanine	-1.6961966	0.000615294	0.004399349
6-deoxyglucitol	2.56397044	0.000721027	0.004909848
phosphogluconic acid	2.806265587	0.00089515	0.005818473
xylulose	2.639000479	0.000983477	0.006014966
guanosine	2.411658511	0.001009505	0.006014966
N-acetylornithine	-1.056675429	0.001121382	0.006414307

5-methoxytryptamine	-1.309372236	0.001458432	0.008021374
fructose-1,6-bisphosphate	-1.305384148	0.001767298	0.009360131
threitol	1.875859851	0.00207681	0.010606567
lactobionic acid	-0.688634365	0.003339298	0.015528642
ethanol phosphate	0.988226466	0.003549848	0.015528642
z C12 FAME with contaminant ions	1.789258514	0.003633288	0.015528642
hydroxylamine	0.822157018	0.003659074	0.015528642
conduritol-beta-expoxide	2.345880599	0.003701919	0.015528642
quinic acid	0.870886944	0.003718876	0.015528642
tyrosine	-1.180245433	0.003900004	0.015528642
glucose-1-phosphate	2.256903328	0.003909309	0.015528642
isothreonic acid	1.600095845	0.004735079	0.018300439
galactose	2.188747236	0.005375894	0.019751312
noradrenaline	-0.85274271	0.005386721	0.019751312
tagatose	2.462831423	0.005864804	0.020966673
glucose	2.032434918	0.007226247	0.02520374
3-deoxyhexitol	1.582591834	0.007852845	0.026737068
2-monoolein	-0.622863242	0.008263446	0.027480761
lactitol	-0.525283401	0.00854372	0.027767091
glyceric acid	-0.547425843	0.009245714	0.029380825
ribose	2.27286992	0.010584293	0.032903344
melezitose	-1.427150664	0.011148604	0.033920221
lyxitol	1.325156603	0.014544258	0.043329768
fructose	1.496507913	0.015383976	0.044896093
methanolphosphate	1.63107425	0.017210372	0.049221663
3,6-anhydro-D-galactose	2.106427968	0.018101946	0.050756437
trehalose-6-phosphate	0.641840271	0.020488031	0.056342086
putrescine	-1.200474728	0.025137183	0.067822967
4-hydroxybutyric acid	1.350670059	0.025848488	0.068450626
tryptophan	-1.458158533	0.029970216	0.07667514
3-hydroxypropionic acid	1.406304519	0.030026628	0.07667514
1-methylgalactose	-0.760033235	0.031875857	0.079106738
uracil	0.501566188	0.03208525	0.079106738

pinitol	1.251192116	0.036607912	0.088727652
glycerol-alpha-phosphate	0.773508265	0.042230601	0.099263899
1-monopalmitin	0.433376433	0.042343342	0.099263899
digitoxose	1.292527319	0.047815143	0.110283314
ribose-5-phosphate	0.982386442	0.049869812	0.113196557
leucine	1.385944082	0.057339766	0.128118539
butane-2,3-diol	1.56640371	0.061543367	0.134913311
adenosine	-0.696136235	0.062267682	0.134913311
citric acid	-0.584363555	0.063269203	0.135037254
stearic acid	0.432781038	0.07381995	0.155239013
N-methylalanine	0.55183218	0.076143251	0.157804129
fumaric acid	-0.888120786	0.078148051	0.159645305
2-isopropylmalic acid	-0.780508261	0.092856252	0.18702034
ketohexose	2.983546032	0.096707592	0.190582971
UDP-N-acetylglucosamine	0.391814231	0.097290608	0.190582971
lactic acid	1.341608393	0.106363758	0.205540775
adenine	-1.075408379	0.110762509	0.211187184
3-aminoisobutyric acid	0.323429841	0.114545854	0.214087459
lactose	-0.69312291	0.115277862	0.214087459
hexose	-2.052366682	0.130207647	0.238714019
methionine	-0.543241327	0.131989387	0.238917498
ethanolamine	0.970653487	0.137932139	0.246553699
phenylalanine	-0.831039596	0.145137546	0.256092709
phosphoenolpyruvate	-0.583572506	0.147173231	0.256092709
inositol-4-monophosphate	0.468742899	0.148668901	0.256092709
glycolic acid	1.077374842	0.151168036	0.256092709
beta alanine	0.416465433	0.152222939	0.256092709
spermidine	0.985975694	0.167454107	0.278441131
maltitol	-0.429965489	0.192994445	0.314454252
trans-4-hydroxy-L-proline	0.530552022	0.193510309	0.314454252
linoleic acid	0.451635766	0.204321516	0.328291875
beta-glutamic acid	-0.313252021	0.221448287	0.351856723
tranexamic acid	0.63724663	0.247225249	0.38849682

maltotriose	-0.334418142	0.250198383	0.388895312
ornithine	0.726611419	0.274849996	0.422618811
fructose-6-phosphate	0.348208715	0.295773296	0.449952993
threonine	-0.253532537	0.308895258	0.464968651
phosphohexonic acid	0.4293565	0.335268092	0.499409762
palmitoleic acid	0.173402624	0.351363656	0.51798972
isothreitol	-0.412585285	0.376724532	0.545877453
O-succinylhomoserine	0.223677195	0.37791516	0.545877453
valine	-0.576292508	0.403246109	0.576641936
malate	0.387526663	0.414448447	0.586793345
glutamine	0.753596046	0.431486468	0.60387952
5'-deoxy-5'-methylthioadenosine	-0.36719127	0.438848493	0.60387952
z hexose pertms	-0.51696785	0.439185106	0.60387952
hexose-6-phosphate	-0.432065112	0.459234335	0.625433427
4-hydroxyproline	0.375980234	0.48637607	0.650073719
tyrosol	-0.139486257	0.486418797	0.650073719
aspartic acid	-0.48359211	0.494482888	0.653408176
pyrophosphate1	0.516904311	0.498052386	0.653408176
maltotriitol	-0.30527346	0.513564728	0.667634147
isoleucine	-0.314373103	0.518263621	0.667672953
aspartate	-0.1358283	0.531384255	0.678463826
galactinol	0.629496368	0.550001715	0.692886498
galactose-6-phosphate	-0.368718127	0.552371054	0.692886498
L-DOPA	-0.45815655	0.587053478	0.722106328
4-aminobutyric acid	0.432314357	0.591851465	0.722106328
glycerol-3-galactoside	0.187149211	0.592388226	0.722106328
serine	0.28018936	0.595863963	0.722106328
alanine	0.408808482	0.620173235	0.74525019
gamma-aminobutyric acid	-0.319805117	0.631463581	0.752494101
inulotriose	0.471063609	0.661244957	0.781471313
urea	0.513508124	0.667926396	0.782897333
oleic acid	0.14388256	0.707845021	0.822941773
glutathione	-0.202080264	0.725834303	0.83705085

3-phosphoglycerate	-0.096805865	0.744996384	0.848042307
oxoproline	-0.23064639	0.762456357	0.848042307
glucose-6-phosphate	0.132940713	0.767108962	0.848042307
phosphoethanolamine	-0.176871051	0.767707687	0.848042307
1,5-anhydroglucitol	0.141134065	0.769449013	0.848042307
citrulline	-0.158005355	0.770947552	0.848042307
cytidine-5-monophosphate	-0.109373495	0.804120325	0.869807729
isohexonic acid	-0.10196825	0.807473527	0.869807729
asparagine	-0.138682666	0.808982014	0.869807729
trans-4-hydroxyproline	-0.097139266	0.819800682	0.872193552
glutamic acid	0.118220955	0.823399507	0.872193552
homoserine	0.045516291	0.857834243	0.901987476
palmitic acid	0.085997521	0.871835759	0.907690941
allothreonine	0.094676208	0.875953495	0.907690941
succinic acid	0.066211159	0.909721619	0.927965522
methylhexose	0.08102231	0.913421308	0.927965522
glycine	0.08140467	0.914986983	0.927965522
lignoceric acid	-0.009126877	0.990812645	0.996962784
2-methylglyceric acid	-0.001239756	0.996962784	0.996962784

Table S3. KEGG pathway impact analysis of differentially metabolite *in S. mutans*

pathway	P value	log_2 (P value)	Impact
Streptomycin biosynthesis	0.046402	4.429669	0
Amino sugar and nucleotide sugar metabolism	0.058465	4.096283	0.05298
Synthesis and degradation of ketone bodies	0.18525	2.432455	0
Inositol phosphate metabolism	0.21814	2.196674	1
Fructose and mannose metabolism	0.30937	1.692595	0.14583
Butanoate metabolism	0.52605	0.926728	0
Aminoacyl-tRNA biosynthesis	0.5519	0.857521	0

Starch and sucrose metabolism	0.58255	0.779546	0.2807
Glycolysis / Gluconeogenesis	0.69046	0.53437	0
Purine metabolism	0.87694	0.18945	0

Table S4. KEGG pathway impact analysis of differentially metabolite in *C. albicans*

PATHWAY	P value	log_2 (P value)	Impact
Amino sugar and nucleotide sugar metabolism	0.002228	8.80997	0.14185
Galactose metabolism	0.007312	7.095558	0.4359
Fructose and mannose metabolism	0.045099	4.470761	0.15686
Ubiquinone and other terpenoid-quinone biosynthesis	0.049292	4.342503	0
Pentose and glucuronate interconversions	0.26291	1.927359	0.27273
Lysine degradation	0.31749	1.655217	0
Tyrosine metabolism	0.31749	1.655217	0
Starch and sucrose metabolism	0.31749	1.655217	0.38071
Aminoacyl-tRNA biosynthesis	0.3201	1.643405	0
Lysine biosynthesis	0.33481	1.578585	0
Pentose phosphate pathway	0.36819	1.441478	0
Arginine biosynthesis	0.36819	1.441478	0.12727
Phenylalanine, tyrosine and tryptophan biosynthesis	0.41529	1.267809	0
Inositol phosphate metabolism	0.43023	1.21682	0.11609
Phosphatidylinositol signaling system	0.48643	1.039696	0.04493
Purine metabolism	0.80283	0.316834	0

Table S5. Differentially expressed genes between control group and treat group

gene_id	Gene.Name	Reads (Treat)	Reads (Control)	log2FoldChange	pval	padj

3634765	YTM1	1654.641462	2437.748857	-0.55903	2.77E-06	4.40E-05
3634767	RAM2	1059.349696	1331.149094	-0.32949	0.009539	0.044235
3634770	HST1	269.7934634	382.9370979	-0.50525	0.003998	0.022418
3634772	NA	679.1014053	500.9500402	0.43896	0.004995	0.026577
3634773	CAALFM_C109070WA	5197.693118	3678.221398	0.49886	0.001187	0.008548
3634774	NA	1374.868266	675.9029932	1.0244	3.07E-09	8.02E-08
3634778	NA	248.9633746	169.3960752	0.55553	0.006483	0.032564
3634780	CAALFM_C110140CA	7866.108637	3663.203963	1.1025	2.12E-15	1.01E-13
3634783	CAALFM_C110170WA	1931.95271	301.1044477	2.6817	0.00065	0.005214
3634786	NA	1927.924704	2779.374791	-0.52771	5.09E-06	7.77E-05
3634788	NA	2058.673533	3333.089681	-0.69515	2.33E-09	6.18E-08
3634789	CAALFM_C108780WA	6295.252776	5145.406802	0.29098	0.006429	0.032367
3634793	CAALFM_C108820CA	254.5040789	357.6245683	-0.49076	0.006572	0.03296
3634812	NA	7397.890671	3106.952107	1.2516	0.000494	0.00415
3634814	NA	373.0398211	514.2220856	-0.46306	0.003688	0.020907
3634824	SHY1	907.2745732	1251.165533	-0.46366	0.00037	0.003275
3634826	CRP1	8066.193804	14603.00319	-0.85631	7.66E-16	3.81E-14
3634831	NA	33.27240724	8.279098285	2.0068	0.001343	0.009482
3634838	CBK1	942.9571641	1209.261455	-0.35886	0.005131	0.027186
3634840	CAALFM_C110410WA	121.8664931	326.6690861	-1.4225	3.09E-12	1.14E-10
3634852	FTH1	846.5291962	1429.1719	-0.75555	1.27E-07	2.60E-06
3634857	CAALFM_C109440WA	3556.532049	1227.336673	1.5349	8.22E-17	4.35E-15

3634858	NA	3863.8827	4728.413477	-0.2913	0.009213	0.043112
3634863	CAALFM_C109980CA	1813.329964	815.2966767	1.1532	1.06E-19	6.66E-18
3634864	SAC1	1639.582629	1202.636429	0.44713	0.000325	0.002936
3634868	DBP3	1814.560847	2567.121547	-0.50053	1.82E-05	0.000236
3634875	MLS1	151198.4434	67991.57339	1.153	7.24E-28	6.69E-26
3634876	NA	90.01003854	31.23570017	1.5269	0.006038	0.03097
3634881	NA	961.8338928	584.7485845	0.71797	1.93E-07	3.85E-06
3634883	RPA34	359.0680042	502.0700383	-0.48363	0.002788	0.016778
3634892	CAALFM_C109620CA	337.3606903	526.4312248	-0.64195	6.89E-05	0.000758
3634901	NA	338.32806	579.2215633	-0.77569	1.09E-06	1.89E-05
3634902	CAALFM_C110280CA	2612.099109	1320.546545	0.98407	0.001528	0.010518
3634904	MNN12	2207.294799	2808.677948	-0.34761	0.002792	0.016787
3634906	CAALFM_C110310WA	861.3582971	445.3531536	0.95166	7.38E-11	2.28E-09
3634919	CAALFM_C108840WA	392.8990801	571.3597341	-0.54024	0.00558	0.029073
3634928	NA	1487.01558	2041.368437	-0.45712	0.000141	0.00143
3634934	NA	1032.32531	1403.890975	-0.44353	0.000408	0.003523
3634940	NA	1105.434561	786.192031	0.49166	0.000218	0.002079
3634945	AOX2	1939.976441	7808.626476	-2.009	6.89E-45	1.19E-42
3635141	NA	440.6222087	307.9218694	0.51698	0.002435	0.015158
3635147	NA	1669.252531	2478.445987	-0.57023	1.31E-06	2.22E-05
3635152	STP1	2315.938813	1248.325633	0.8916	2.13E-13	8.83E-12
3635172	NA	3334.740069	4300.243911	-0.36684	0.001161	0.008376

3635189	CAALFM_C304630WA	3542.440267	1980.330604	0.839	2.48E-13	1.01E-11
3635197	SAP3	497.7533802	225.3012889	1.1436	2.91E-11	9.60E-10
3635203	CAALFM_C305290CA	503.187459	269.1651447	0.9026	8.98E-08	1.88E-06
3635222	FAS2	75788.41194	114265.8445	-0.59235	1.63E-08	3.78E-07
3635229	CAALFM_C305160CA	7599.344031	10122.61414	-0.41364	0.000407	0.003522
3635240	TRY4	4119.521417	1868.920695	1.1403	2.76E-23	2.08E-21
3635241	NA	286.8841004	185.1408534	0.63184	0.001102	0.008052
3635255	SEN1	3717.91387	4646.0485	-0.32151	0.004121	0.022889
3635261	TEC1	5120.794972	7109.652697	-0.47341	0.004279	0.023529
3635265	CDR2	1186.635107	1652.232319	-0.47754	0.000112	0.001172
3635266	NOP14	1931.512048	2494.577304	-0.36906	0.001597	0.010914
3635268	CAALFM_C304920CA	4038.179963	3169.285582	0.34955	0.001798	0.012002
3635269	HGT4	3038.32614	2417.465246	0.32978	0.003743	0.021147
3635271	ARF2	6982.262046	5663.223366	0.30207	0.005694	0.029504
3635280	SBP1	18935.99312	14535.50272	0.38155	0.000745	0.005856
3635584	CAALFM_C401760WA	445.0936982	897.5562065	-1.0119	4.25E-11	1.35E-09
3635587	TIM12	146.3341886	234.4763858	-0.68018	0.001275	0.009099
3635593	CAALFM_C401830CA	737.0664597	539.6233111	0.44984	0.001637	0.011081
3635594	CAALFM_C401820CA	1496.213832	1967.449466	-0.39501	0.001103	0.008052
3635595	CAALFM_C401800WA	365.9916993	87.61702231	2.0625	0.000352	0.003148
3635598	ILV6	3344.392504	2628.222588	0.34766	0.002204	0.014066
3635599	PGA53	1233.84901	848.6950352	0.53985	2.90E-05	0.00036

3635605	CAALFM_C401300WA	2866.362256	3628.548555	-0.34017	0.002276	0.014386
3635614	MDN1	16032.83244	23001.68199	-0.52071	8.80E-07	1.56E-05
3635618	NIP1	5119.305037	6215.09456	-0.27983	0.009857	0.045438
3635623	PWP1	1787.227465	2415.441387	-0.43456	0.000262	0.002415
3635636	MDH1-1	11818.14722	9687.690211	0.28678	0.007172	0.035346
3635642	NA	907.522926	1178.644157	-0.37712	0.004207	0.023248
3635643	NA	200.0947271	128.6470946	0.63726	0.00458	0.024856
3635647	HGH1	938.7973107	1226.857944	-0.38608	0.002924	0.017408
3635651	AAT22	1306.091417	442.5448582	1.5614	3.42E-29	3.34E-27
3635659	CAT2	23297.44464	7920.286747	1.5565	4.89E-47	1.07E-44
3635661	RGA2	6619.991178	5435.610136	0.28439	0.009548	0.044235
3635667	CAALFM_C402170CA	1002.773733	776.4547509	0.36902	0.006218	0.031545
3635668	NA	1900.146198	1353.794748	0.4891	5.72E-05	0.000641
3635671	CYT2	742.7090091	945.150718	-0.34775	0.009826	0.045363
3635674	PTC8	1889.631198	3208.14426	-0.76363	2.17E-06	3.55E-05
3635687	HGT17	21253.98801	770.120427	4.7865	#######	#######
3635693	CAALFM_C402230CA	113.9624075	32.21777356	1.8226	5.42E-08	1.16E-06
3635694	ZCF25	298.4409533	151.913771	0.97419	0.000484	0.004079
3635699	CAALFM_C401290WA	3542.944712	4367.200546	-0.30176	0.006395	0.032244
3635700	CAALFM_C401280CA	1230.765292	1719.63608	-0.48255	0.000507	0.004233
3635704	NAT4	167.3411064	251.3165477	-0.58671	0.003809	0.02145
3635714	CAALFM_C401560CA	1472.555298	1132.224988	0.37916	0.002714	0.016439

3635716	ERG251	4159.393363	2261.929762	0.87882	8.96E-15	4.08E-13
3635717	NA	764.0310232	562.5781656	0.44158	0.002029	0.01327
3635727	ESC4	105.0827119	54.72550249	0.94124	0.002565	0.015798
3635733	NA	417.240209	725.0953586	-0.79729	5.34E-06	8.06E-05
3635742	NA	2370.851791	1319.840322	0.84504	1.91E-12	7.16E-11
3635755	CAALFM_C202410WA	5249.116818	4183.786583	0.32727	0.002581	0.015855
3635756	CAALFM_C202420CA	2825.279868	3671.517286	-0.37798	0.00095	0.007144
3635757	UTP21	3198.202683	4158.563774	-0.37882	0.004714	0.02543
3635764	SRD1	562.4267387	307.2762323	0.87213	5.98E-08	1.28E-06
3635770	ARO3	2206.9385	1657.252502	0.41325	0.000593	0.004818
3635775	CAALFM_C202230CA	792.9351064	93.20200922	3.0888	1.67E-63	4.97E-61
3635777	OPI1	1125.577275	737.1648257	0.61061	1.21E-05	0.000167
3635781	NA	4892.552101	6093.242021	-0.31662	0.00308	0.018135
3635783	NA	913.1338339	1168.682003	-0.35598	0.006296	0.031819
3635784	COX13	13833.67278	11339.77237	0.28679	0.006286	0.031792
3635788	CTF1	2499.006187	1828.189339	0.45094	0.000138	0.001406
3635793	CAALFM_C201930CA	894.9059184	1266.06791	-0.50055	9.40E-05	0.001006
3635804	CAALFM_C201540WA	4982.858296	1972.728747	1.3368	9.51E-17	4.95E-15
3635805	CAALFM_C202270CA	499.9784943	726.5860181	-0.53927	0.000185	0.001788
3635806	NA	1026.87129	1307.654923	-0.34873	0.005525	0.028825
3635817	PRS1	2984.659206	4065.76403	-0.44596	6.72E-05	0.000744
3635820	CAALFM_C202540WA	5423.866624	7623.700885	-0.49117	6.08E-06	9.09E-05

3635836	CAALFM_C201870CA	1711.041415	1253.771822	0.4486	0.000325	0.002936
3635837	ZCF6	1436.092547	1058.611723	0.43998	0.00495	0.026423
3635843	NA	5348.969642	2978.422691	0.84471	1.57E-06	2.62E-05
3635847	SPT5	2156.510033	2834.983909	-0.39464	0.003482	0.020038
3635852	FMO1	2702.161231	489.1063201	2.4659	8.11E-80	4.09E-77
3635860	CAALFM_C202580WA	6550.03553	10498.20149	-0.68057	2.81E-10	8.15E-09
3635862	NA	259.7578685	180.734704	0.52329	0.008709	0.04116
3635863	HGT20	178.4974989	100.3857238	0.83035	0.000718	0.005696
3636118	BNI1	2460.177914	3158.388837	-0.36043	0.001644	0.011115
3636120	NA	2570.625822	3218.719573	-0.32437	0.004592	0.024856
3636123	CAALFM_C113060CA	3375.454958	4382.49383	-0.37667	0.000719	0.005699
3636125	NA	668.6150874	22.56394726	4.8891	2.68E-35	3.09E-33
3636132	MET3	22361.03493	30088.03948	-0.4282	3.76E-05	0.000448
3636141	NA	394.3886965	527.5802058	-0.41977	0.006799	0.033919
3636144	CAALFM_C113270WA	1601.282203	986.0692273	0.69947	0.000138	0.001406
3636154	NA	561.769685	887.814558	-0.66028	2.66E-06	4.24E-05
3636176	CAALFM_C112830CA	2886.807045	2069.042137	0.48051	3.78E-05	0.00045
3636179	CAALFM_C112860CA	426.3834182	615.7049404	-0.53009	0.000548	0.004518
3636180	NA	109.235645	196.2546089	-0.84528	0.000343	0.003076
3636193	OSM2	65959.64089	46304.5082	0.51043	0.005297	0.027822
3636203	TYE7	9053.163646	12985.19878	-0.52037	1.54E-05	0.000205
3636211	KIS2	4752.723027	3629.192445	0.38911	0.000457	0.003884

3636213	CYB2	33287.56336	23367.49901	0.51048	9.29E-07	1.64E-05
3636219	NA	5368.525324	3315.896483	0.69513	0.002835	0.01697
3636225	OFI1	11.32152092	48.15844219	-2.0887	5.66E-05	0.000636
3636226	NA	5674.088062	10023.46317	-0.82092	1.35E-05	0.000184
3636228	KNS1	3998.457165	2717.354884	0.55724	7.45E-07	1.33E-05
3636229	HSP70	287192.5873	152184.6214	0.9162	9.16E-07	1.62E-05
3636393	CAALFM_C504610WA	1507.523653	2043.726112	-0.43902	0.000268	0.002452
3636394	CAALFM_C504620CA	738.0420698	1021.314054	-0.46865	0.001385	0.009739
3636397	CAALFM_C504640CA	1711.403314	2161.266441	-0.3367	0.004239	0.023389
3636406	PGA4	3984.184092	3170.823723	0.32943	0.003352	0.019478
3636411	NA	1928.281822	1448.093888	0.41316	0.000656	0.005251
3636413	NA	8876.079641	2993.086216	1.5683	6.51E-45	1.15E-42
3636414	CAALFM_C504470CA	13257.91198	8756.634567	0.59841	0.000142	0.001432
3636416	CAALFM_C504900CA	428.3415586	575.9652687	-0.42722	0.00542	0.028333
3636422	NA	2234.833192	1654.981775	0.43335	0.000249	0.002311
3636429	NA	1452.947852	984.4704997	0.56156	9.99E-06	0.000141
3636434	SFL2	803.9848733	1099.531106	-0.45165	0.000653	0.005234
3636437	CAALFM_C504860CA	291.2957667	154.8582438	0.91154	7.95E-06	0.000116
3636439	PUT2	28296.34334	38670.51077	-0.45062	2.05E-05	0.000261
3636443	HAS1	5251.748493	6807.283337	-0.37428	0.000622	0.005024
3636448	PFK1	10282.84934	13677.46568	-0.41156	0.002326	0.014635
3636450	NA	76.63545224	189.5180199	-1.3063	1.37E-07	2.79E-06

3636453	GLN3	2057.596897	1593.324667	0.36892	0.002101	0.013579
3636458	CCT3	4123.069699	3375.102701	0.28879	0.010194	0.046635
3636460	CAALFM_C505140WA	467.0211833	629.2861866	-0.43023	0.004063	0.022666
3636465	CAALFM_C505180WA	126.4398277	63.98667416	0.98261	0.000665	0.005307
3636467	NA	1018.043177	764.0242331	0.41411	0.002079	0.013511
3636468	CAALFM_C505210WA	1647.875474	1278.946427	0.36565	0.003174	0.018607
3636472	NA	924.1753603	1263.762084	-0.45149	0.000387	0.003388
3636477	CAALFM_C504310WA	810.9298297	592.8001885	0.45203	0.003571	0.020394
3636483	NA	1586.903006	2118.996071	-0.41717	0.000447	0.003812
3636484	PGA37	319.4712723	735.7944661	-1.2036	5.54E-05	0.000629
3636485	CAALFM_C504380CA	164.5607837	12.60092015	3.707	8.30E-16	4.09E-14
3636489	ADH1	61719.90655	107933.0014	-0.80633	8.04E-11	2.45E-09
3636491	NA	1593.906465	1250.043787	0.35059	0.004589	0.024856
3636498	MRP2	861.1346657	1136.46423	-0.40024	0.002128	0.013716
3636499	NA	3338.875201	2600.108753	0.36079	0.001396	0.009797
3636503	CAR1	15620.09914	7875.050936	0.98804	4.55E-20	3.01E-18
3636507	CAALFM_C504530WA	955.4345843	1391.400744	-0.54231	1.91E-05	0.000245
3636521	SCW11	214.9589297	131.4817518	0.7092	0.008088	0.038814
3636523	CHT2	338.3198195	205.711671	0.71776	0.000144	0.001453
3636526	EST1	393.9799955	533.6166867	-0.43768	0.005691	0.029504
3636529	DES1	399.7842133	597.800125	-0.58044	0.000176	0.001714
3636535	PPR1	1032.416774	787.792506	0.39014	0.003541	0.020254

3636552	ILV2	7562.31733	5231.186244	0.53169	1.04E-06	1.83E-05
3636555	NA	356.3977672	653.1132989	-0.87384	1.73E-08	3.97E-07
3636558	PXP2	9234.033448	485.4318827	4.2496	#######	#######
3636565	CAALFM_C301850WA	838.7048749	1231.289581	-0.55393	0.002334	0.014666
3636568	NA	11273.31342	8112.570823	0.47468	9.79E-06	0.000139
3636578	CAALFM_C302060WA	238.5892822	124.9138178	0.9336	2.24E-05	0.000283
3636592	CAALFM_C302020WA	2664.761932	3459.065532	-0.37638	0.001066	0.00784
3636598	POX1-3	80980.49415	23030.37826	1.814	2.67E-64	8.34E-62
3636605	CAALFM_C301680CA	638.6487097	847.346669	-0.40793	0.003303	0.019208
3636618	NA	634.8191133	889.3017226	-0.48633	0.000494	0.00415
3636623	NA	757.8848479	570.6886089	0.40927	0.004045	0.0226
3636625	NA	187.8058365	282.4679203	-0.58885	0.00288	0.017192
3636627	PMC1	14468.3335	10667.31108	0.4397	3.97E-05	0.000468
3636628	ARF3	858.3508829	539.8174542	0.6691	2.40E-06	3.89E-05
3636629	FOX3	24441.501	6674.877477	1.8725	2.70E-66	9.32E-64
3636634	NA	13894.13338	2632.745543	2.3998	#######	5.39E-97
3636640	CAP1	12710.13405	9606.454994	0.4039	0.001117	0.008107
3636646	GEA2	3290.510999	2649.185187	0.31276	0.006991	0.034609
3636651	ZCF8	1141.981356	901.933373	0.34045	0.010574	0.047972
3636653	LAP41	4164.757238	2950.379204	0.49733	7.84E-06	0.000115
3636655	ERG6	4555.640995	2293.381598	0.99018	4.64E-09	1.17E-07
3636656	CAALFM_C302140CA	4202.036071	2424.46006	0.79343	3.31E-11	1.07E-09

3636661	CAALFM_C302090CA	921.0145182	607.8158757	0.59959	1.65E-05	0.000217
3636669	CAALFM_C301560WA	3989.602191	4993.958655	-0.32394	0.003522	0.020197
3636793	CAALFM_C102410CA	445.8408965	324.0294457	0.4604	0.006419	0.032341
3636797	SCW4	147.2547558	7.072151577	4.38	5.34E-10	1.49E-08
3636798	DIP5	48847.99932	32437.46654	0.59064	1.76E-08	4.04E-07
3636801	PUT4	1223.220965	218.9002626	2.4823	3.08E-11	1.01E-09
3636802	HNM4	1352.024174	346.5925193	1.9638	4.01E-11	1.28E-09
3636811	ARX1	2839.518659	4361.6393	-0.61922	3.76E-08	8.28E-07
3636815	HOM6	1194.08369	866.4048338	0.46279	0.000343	0.003076
3636816	EXG2	469.5215115	804.2997263	-0.77654	0.003496	0.020097
3636835	CAALFM_C102970WA	3178.410987	2220.256673	0.51758	6.34E-06	9.43E-05
3636836	GOR1	647.3749775	301.4391366	1.1027	3.83E-12	1.39E-10
3636837	XOG1	521.407134	300.8488442	0.79337	1.36E-06	2.29E-05
3636858	EHD3	12032.91334	5478.274034	1.1352	1.18E-25	1.02E-23
3636867	NA	1110.625067	1401.81177	-0.33592	0.007499	0.036544
3636871	CAALFM_C102780WA	4609.984423	3710.190557	0.31327	0.0044	0.024042
3636873	CAALFM_C102800WA	2978.593114	1229.023223	1.2771	0.000883	0.006735
3636875	LYS2	6936.613245	5484.748904	0.33881	0.001911	0.012638
3636881	NA	429.7024685	566.7032233	-0.39926	0.01002	0.046077
3636885	DAO1	5950.030842	4187.308243	0.50687	3.96E-06	6.11E-05
3636889	CAALFM_C103360WA	3136.099857	3984.627993	-0.34547	0.002217	0.014118
3636901	NA	2713.83295	3417.032266	-0.33241	0.003622	0.02062

3636904	NA	3815.013552	5024.772718	-0.39737	0.000278	0.002537
3636913	AGE1	1243.58813	1848.401727	-0.57177	2.55E-06	4.09E-05
3636914	CAALFM_C103490WA	3605.85324	5999.748284	-0.73456	2.11E-10	6.18E-09
3636915	YPT1	6650.638011	5369.55212	0.30869	0.004582	0.024856
3636918	MED17	1737.63024	2300.569681	-0.40487	0.000592	0.004815
3636933	CAALFM_C702340CA	740.7673496	977.0575434	-0.39942	0.003398	0.019693
3636935	SEC1	1861.631702	1467.599178	0.34311	0.004359	0.023908
3636936	CAALFM_C702310CA	344.4742353	204.6733793	0.75107	6.20E-05	0.000688
3636942	DPP3	440.9221442	302.7567726	0.54236	0.009461	0.044015
3636943	PEX1	11610.8549	4676.382085	1.312	3.57E-33	3.90E-31
3636948	EXO70	1644.689911	1183.669581	0.47455	0.000138	0.001406
3636950	CUP9	9257.590775	11620.55811	-0.32797	0.003157	0.018539
3636951	HSP90	108067.3894	63379.66347	0.76984	0.000394	0.003436
3636954	CAALFM_C702010CA	1135.057661	165.2991267	2.7796	5.40E-68	2.09E-65
3636958	CAALFM_C701460CA	301.4028854	178.3199369	0.75723	0.010096	0.046281
3636960	NA	385.6823697	533.4181752	-0.46785	0.002783	0.016765
3636968	TOM40	3834.957356	4836.242013	-0.33468	0.00253	0.015669
3636972	NA	1914.78809	1048.509897	0.86884	1.93E-12	7.18E-11
3636977	AHP2	3305.746661	1570.59556	1.0737	4.93E-20	3.23E-18
3636978	CYP1	27761.3058	11617.11277	1.2568	1.20E-23	9.14E-22
3636982	NA	583.8822397	818.4703913	-0.48725	0.00071	0.005637
3636987	NA	81.46059982	35.7534125	1.188	0.001144	0.008284

3636996	CAALFM_C702140WA	21.03855951	4.378040199	2.2647	0.006462	0.032509
3636999	NA	1006.935727	396.5490743	1.3444	0.001628	0.01103
3637001	NA	436.2725055	145.0928545	1.5883	4.41E-05	0.000516
3637002	NA	1422.127651	603.3587501	1.237	1.50E-05	0.0002
3637004	FMP45	7758.945179	6307.23279	0.29885	0.006057	0.031018
3637013	NA	2187.856761	1175.814742	0.89586	3.41E-05	0.000411
3637019	CAALFM_C702120CA	494.0607308	658.0227922	-0.41345	0.005019	0.026679
3637036	CAALFM_C701600WA	522.2527174	822.424173	-0.65513	4.94E-06	7.55E-05
3637037	CAALFM_C701590CA	752.6111172	955.1673424	-0.34385	0.010347	0.047102
3637041	NUP	91.64962264	43.52829319	1.0742	0.001221	0.008763
3637045	ALK6	183.9047163	93.98731846	0.96842	7.61E-05	0.000833
3637323	PYC2	33830.56686	8184.639831	2.0473	1.27E-78	5.94E-76
3637326	CAALFM_C204280WA	193.6850382	123.2861073	0.6517	0.004254	0.023434
3637329	UGA1	14166.22744	8849.241193	0.67883	1.66E-10	4.90E-09
3637337	CAALFM_C203560CA	603.1030658	899.4307837	-0.57661	0.000205	0.001966
3637338	CAALFM_C203550CA	1854.474815	2409.547199	-0.37775	0.001289	0.009182
3637342	ADAEC	21024.69035	11128.53995	0.91782	4.65E-05	0.000539
3637356	BAT21	1447.170496	1101.914269	0.39322	0.007809	0.037748
3637364	NA	273287.0283	156510.2368	0.80416	0.000163	0.001616
3637365	NA	645.6673297	114.8682106	2.4908	2.20E-13	9.09E-12
3637367	GCD7	425.8212894	600.9422357	-0.49698	0.001305	0.009269
3637371	CAALFM_C203760CA	5923.585065	4231.651702	0.48525	9.83E-06	0.000139

3637372	YIM1	5756.015046	3425.450163	0.74878	4.81E-10	1.35E-08
3637374	NA	171.7493126	69.90809736	1.2968	2.93E-06	4.61E-05
3637378	CAALFM_C204110WA	481.9569097	888.20896	-0.88199	8.19E-10	2.25E-08
3637391	NA	288.8422408	42.26512816	2.7727	4.64E-28	4.35E-26
3637399	NA	625.6325619	965.8049895	-0.62642	5.20E-06	7.90E-05
3637401	CAALFM_C203800CA	503.7144861	667.87513	-0.40697	0.005261	0.027744
3637414	HSP78	31563.65003	14227.89134	1.1495	0.003024	0.017855
3637418	MED1	1536.380433	616.2109054	1.318	1.68E-05	0.000221
3637419	NA	270.8158759	384.4532454	-0.5055	0.004021	0.022525
3637420	CAALFM_C203950WA	503.0739132	656.2835187	-0.38355	0.009329	0.043495
3637429	NA	836.3999966	608.4931167	0.45895	0.00097	0.007275
3637430	NA	6937.549473	5272.701162	0.39588	0.000242	0.002248
3637431	NA	11252.31771	14446.76657	-0.36052	0.00901	0.042338
3637433	AVT7	2836.602209	3841.777756	-0.43761	9.01E-05	0.000971
3637437	UGA11	2295.334996	1649.898385	0.47633	5.41E-05	0.000617
3637701	CAALFM_C503080CA	4272.838304	2355.516562	0.85915	3.94E-09	1.02E-07
3637705	CAALFM_C503120WA	390.3623299	280.7558823	0.4755	0.005808	0.029975
3637711	NA	3894.325342	4746.835615	-0.28559	0.010295	0.046932
3637718	UTP13	2149.551236	2958.393088	-0.46078	0.000596	0.004837
3637731	NA	800.3424863	506.2530618	0.66076	5.54E-06	8.32E-05
3637739	CAALFM_C502740WA	1194.425647	1555.490303	-0.38105	0.002093	0.013543
3637743	MSW1	257.3078027	357.3707121	-0.47393	0.007745	0.037497

3637744	GRP2	4839.449436	1891.376574	1.3554	1.62E-06	2.70E-05
3637751	CAALFM_C502920WA	1351.55433	992.2954829	0.44578	0.000515	0.004281
3637757	LEU3	3112.413141	3952.802874	-0.34484	0.00206	0.013419
3637758	NA	45.7697685	98.95477743	-1.1124	0.000602	0.004878
3637770	IFH1	4331.23842	5835.543819	-0.43009	7.60E-05	0.000833
3637775	CAALFM_C502690WA	5205.263987	1601.604639	1.7005	9.20E-49	2.08E-46
3637785	CAALFM_C502640WA	342.3310253	679.9138669	-0.98996	1.14E-10	3.40E-09
3637794	CAALFM_C502380WA	17923.71032	12630.96915	0.5049	0.000645	0.005177
3637799	CAALFM_C503150WA	3304.989902	2358.248393	0.48693	0.000148	0.001487
3637806	GAP1	4427.031916	5874.435847	-0.40811	0.001954	0.012845
3637821	CCC2	5402.718799	6810.068916	-0.33398	0.009793	0.045271
3637824	CAALFM_C503040WA	223.2764966	311.8220538	-0.48189	0.010031	0.046081
3637827	TNA1	493.4236181	77.99130518	2.6614	4.40E-39	6.01E-37
3637828	CAALFM_C402680CA	125.3940141	226.0576153	-0.85022	0.000116	0.001206
3637839	NCP1	5971.824021	4729.068724	0.33662	0.001798	0.012002
3637840	NA	260.2181521	176.4972097	0.56008	0.005149	0.027261
3637843	CAALFM_C403140CA	573.481286	812.2099107	-0.50211	0.000764	0.005979
3637844	CAALFM_C402460WA	181.5413351	117.3093395	0.62998	0.007156	0.035297
3637850	AHP1	37539.07387	30578.28879	0.29589	0.009651	0.04465
3637853	CGR1	761.8995138	380.9457156	1	3.05E-11	1.00E-09
3637854	NA	362.6540281	549.1928099	-0.59872	0.000161	0.0016
3637858	GRX3	2622.500563	3501.891969	-0.41719	0.000215	0.002056

3637861	CAALFM_C402690WA	702.6216722	1105.673908	-0.65411	0.000342	0.003076
3637862	ARG83	1178.357423	1517.328239	-0.36476	0.003509	0.020154
3637865	CAALFM_C402510WA	481.3431982	339.2972417	0.50452	0.002081	0.013511
3637866	ADR1	17959.94534	11067.89215	0.6984	4.86E-11	1.54E-09
3637867	ZCF15	2492.599958	1968.399936	0.34063	0.003921	0.022023
3637879	NA	17024.59661	21916.80582	-0.36442	0.000497	0.004163
3637883	NA	6655.650368	4745.741105	0.48795	8.17E-06	0.000119
3637899	NA	619.828344	922.5577879	-0.57377	3.42E-05	0.000413
3637903	CAALFM_C402770CA	2015.455175	1179.118518	0.7734	2.87E-05	0.000357
3637910	CAALFM_C402850WA	2407.771365	3252.317316	-0.43377	0.000162	0.001609
3637911	HCA4	848.9407001	1135.539248	-0.41964	0.001491	0.01032
3637916	CAALFM_C402570CA	295.2718707	439.4317304	-0.5736	0.000761	0.005961
3637926	CAALFM_C403090WA	1371.183857	937.2634007	0.5489	2.29E-05	0.000289
3637930	CAALFM_C403050CA	3964.463101	2873.42357	0.46435	3.84E-05	0.000455
3637934	CAALFM_C403020WA	5182.821995	6443.547571	-0.31412	0.003405	0.019699
3637937	GST2	4133.827771	2421.866154	0.77136	1.34E-11	4.54E-10
3637951	PHM5	2472.266575	3098.101215	-0.32555	0.004387	0.024035
3637954	YML6	650.7291295	906.4467169	-0.47817	0.000977	0.007315
3637956	GPH1	48709.21916	67293.6801	-0.46628	6.01E-05	0.00067
3637960	CAALFM_C700760CA	94.99207413	46.05374958	1.0445	0.001564	0.010732
3637971	VPS70	4528.174946	2682.675736	0.75526	1.33E-11	4.54E-10
3637972	CAALFM_C700160CA	708.2889431	915.4847627	-0.3702	0.006946	0.034491

3637985	MAC1	1218.421358	875.4613782	0.4769	0.010672	0.04835
3637993	CAALFM_C700430WA	3634.661078	1637.049724	1.1507	5.08E-23	3.79E-21
3637994	CAALFM_C700590WA	788.4271949	1042.561239	-0.40308	0.002137	0.013743
3637996	RPA135	8679.81501	10980.01151	-0.33914	0.001604	0.010927
3638013	CAALFM_C700060CA	1343.513297	840.8648099	0.67606	1.44E-07	2.92E-06
3638014	RAD3	783.5634854	1005.518299	-0.35982	0.007467	0.036446
3638019	CAALFM_C701030CA	6816.550644	8365.793703	-0.29546	0.005949	0.03056
3638020	CAALFM_C701020CA	417.1301234	615.9254453	-0.56226	0.000894	0.006788
3638028	CAALFM_C700630CA	524.155815	780.5262113	-0.57445	6.96E-05	0.000765
3638029	GUS1	3906.17389	4871.211864	-0.31852	0.004053	0.022628
3638033	NA	669.133874	475.2685967	0.49355	0.000823	0.006342
3638034	LEU2	1329.441775	780.7238491	0.76794	3.96E-09	1.02E-07
3638036	NA	201.1054389	114.4465731	0.81328	0.000378	0.003319
3638038	NA	4397.375353	3370.813356	0.38355	0.003655	0.020778
3638039	NA	53133.06934	14285.73358	1.895	0.000252	0.00233
3638047	HGT13	7146.573658	1258.886816	2.5051	#######	2.27E-97
3638052	CAALFM_C700880CA	2344.51956	3256.956202	-0.47423	3.27E-05	0.000397
3638054	CAALFM_C700870WA	1194.306001	698.0444293	0.77478	2.93E-05	0.000362
3638299	CAALFM_C303560WA	857.6986095	1147.435692	-0.41987	0.001456	0.010097
3638301	GTT13	42.32893195	9.850590448	2.1034	0.000168	0.001659
3638305	FUR4	608.7586361	156.603633	1.9588	1.48E-05	0.000198
3638306	CAALFM_C303470WA	1504.969602	808.1401977	0.89706	0.001414	0.009881

3638307	ALT1	8311.625058	5381.860591	0.62703	6.93E-09	1.69E-07
3638314	ULP1	445.6255056	614.5533384	-0.46371	0.00511	0.027098
3638320	CAALFM_C303260WA	1995.913652	2553.123032	-0.35521	0.002389	0.014953
3638321	NA	331.1980341	495.4748689	-0.58112	0.000277	0.002537
3638325	NA	14353.60824	8908.111697	0.68822	0.000224	0.002125
3638326	CAALFM_C302620CA	997.2412695	553.2045572	0.85013	8.92E-10	2.41E-08
3638333	THI6	652.0583978	896.5399082	-0.45937	0.001814	0.012069
3638334	CAALFM_C302870CA	661.7602756	1054.484044	-0.67216	0.002086	0.013523
3638339	NA	4516.172469	3631.441178	0.31456	0.00494	0.026388
3638346	PHA2	367.4791757	523.6246767	-0.51087	0.001316	0.009319
3638350	CCP1	15705.93845	8961.602143	0.80948	2.64E-08	5.97E-07
3638353	CAALFM_C303410CA	16839.38967	8916.277485	0.91733	4.34E-12	1.56E-10
3638356	CAALFM_C303440CA	237.8338434	106.8693303	1.1541	0.001145	0.008284
3638357	NA	417.3585351	146.1873646	1.5135	1.60E-15	7.71E-14
3638369	PAM17	984.058673	1408.016032	-0.51685	4.27E-05	0.000501
3638375	CAALFM_C302570WA	1094.191484	755.9137898	0.53357	5.98E-05	0.000667
3638386	CAALFM_C302980CA	107.7880507	33.17173778	1.7002	0.001935	0.012771
3638398	USO5	218.6598197	147.757983	0.56545	0.00922	0.043112
3638404	NA	177.8053433	43.52741951	2.0303	1.09E-12	4.18E-11
3638406	SLD1	2733.626786	1960.430912	0.47965	0.00036	0.003202
3638408	SMC3	538.3161615	325.686139	0.72497	8.28E-06	0.00012
3638411	CAALFM_C302750WA	549.9362977	202.848031	1.4389	1.44E-12	5.43E-11

3638418	FAA21	16667.51757	2932.495508	2.5068	#######	#######
3638419	NA	1468.230316	1038.803348	0.49916	6.83E-05	0.000753
3638430	CAALFM_C307290WA	4636.0505	6174.839313	-0.41351	0.000136	0.001397
3638431	NA	1110.737293	1674.539789	-0.59225	0.000227	0.002139
3638437	GCD2	1828.997728	2565.528062	-0.4882	3.54E-05	0.000425
3638441	NA	1193.556663	389.924922	1.614	9.00E-30	9.22E-28
3638444	HHT1	10600.90088	8538.014674	0.31221	0.003584	0.020442
3638445	RRD1	684.1701254	425.7916448	0.68421	5.25E-05	0.000602
3638446	CAALFM_C307070CA	695.5783308	513.3243397	0.43834	0.003225	0.018841
3638450	CAALFM_C307760CA	320.5769091	209.4168386	0.61429	0.001077	0.007909
3638451	NA	3193.606767	4404.716972	-0.46386	3.11E-05	0.000381
3638469	NOP13	1222.530949	1768.724746	-0.53284	1.47E-05	0.000198
3638474	NA	1191.003432	897.0757297	0.40888	0.001926	0.012722
3638477	GCY1	5831.211145	2639.47689	1.1435	1.72E-17	9.40E-16
3638478	CAALFM_C307330WA	838.5982494	583.7673848	0.52259	0.000195	0.001885
3638482	TPI1	7880.132537	10802.2941	-0.45505	2.17E-05	0.000276
3638489	FUM12	13807.27431	10886.56525	0.34288	0.00104	0.007686
3638492	RIT1	624.3782775	901.8481718	-0.53046	0.000144	0.001447
3638504	CAALFM_C306730WA	1022.511206	490.286905	1.0604	7.99E-11	2.45E-09
3638507	NA	2668.199627	1295.754985	1.0421	0.000113	0.001181
3638511	NA	1171.078749	721.5333579	0.6987	1.54E-07	3.12E-06
3638512	TPD3	2628.87515	2082.322045	0.33625	0.003669	0.020839

3638525	CAALFM_C307550CA	2764.804925	3496.754108	-0.33884	0.002683	0.016324
3638528	CAALFM_C307490WA	926.3666929	1229.388642	-0.40829	0.001798	0.012002
3638531	CAALFM_C307230WA	2542.547381	2038.003201	0.31912	0.005878	0.030291
3638534	ZFU2	515.5313924	380.7480778	0.43722	0.006975	0.034568
3638537	NA	109.7310304	41.45083607	1.4045	1.43E-05	0.000194
3638540	TDH3	117983.583	190099.0189	-0.68816	1.65E-05	0.000217
3638791	CHA1	6181.099199	4002.637382	0.62691	1.20E-08	2.85E-07
3638803	NA	592.1950261	768.2907103	-0.37558	0.008841	0.041726
3638820	CAALFM_C200410CA	1104.258721	1507.728884	-0.4493	0.000359	0.0032
3638828	ARO8	3483.868421	2238.129011	0.6384	2.37E-08	5.41E-07
3638834	CAALFM_C201070WA	2584.809569	3426.741437	-0.40678	0.000376	0.003308
3638837	NAT2	2355.618769	3025.700139	-0.36116	0.001761	0.011826
3638840	NDT80	947.6171832	1190.261256	-0.3289	0.010322	0.047024
3638847	CAALFM_C200640WA	1127.44741	1435.854892	-0.34885	0.004968	0.026454
3638848	CAALFM_C200630CA	311.7687372	86.80273022	1.8447	3.50E-10	1.00E-08
3638855	CAALFM_C200550WA	172.7132221	66.56834888	1.3755	1.72E-07	3.44E-06
3638857	HNM1	8362.102468	6535.960776	0.35547	0.000965	0.007254
3638862	PEX12	2221.54397	1653.491116	0.42605	0.000387	0.003388
3638871	PHHB	711.4380846	2703.835972	-1.9262	1.89E-46	3.88E-44
3638873	HGT6	24992.12085	48559.51536	-0.95828	1.62E-08	3.77E-07
3638875	HGT8	23831.49974	33816.57104	-0.50486	1.19E-06	2.03E-05
3638884	CAALFM_C200700WA	9099.438861	11139.92082	-0.29189	0.005274	0.027767

3638905	NA	4363.340268	963.8425901	2.1786	1.21E-29	1.22E-27
3638906	CAALFM_C200170CA	1657.668817	2252.10728	-0.44212	0.000226	0.002137
3638909	NA	1718.690228	1241.820907	0.46885	0.000149	0.001492
3638911	NA	240.3822942	363.967629	-0.59848	0.001411	0.00987
3638913	CAALFM_C200730CA	652.530382	867.7497053	-0.41123	0.003031	0.017878
3638922	UTP5	2121.639244	2920.429536	-0.461	7.92E-05	0.000862
3638925	SIK1	6934.755629	8451.714414	-0.2854	0.007399	0.036194
3638926	UGA3	6331.936837	9458.533749	-0.57897	5.31E-08	1.15E-06
3638933	AHA1	5513.602587	2018.87294	1.4494	2.56E-06	4.10E-05
3638934	CAALFM_CR10280WA	1744.525753	1125.859934	0.63181	9.51E-05	0.001014
3638938	NA	54.02405204	3.788621427	3.8339	8.90E-10	2.41E-08
3638940	PTP3	8008.932286	11759.59773	-0.55416	2.29E-07	4.50E-06
3638950	TRX1	9563.790627	6936.97746	0.46328	0.000367	0.003257
3638957	CAALFM_CR10410CA	1745.927865	2307.924672	-0.4026	0.000662	0.005289
3638966	INO1	4616.049333	13044.65551	-1.4987	8.79E-17	4.61E-15
3638974	CAALFM_CR10420WA	4369.751596	5672.580374	-0.37645	0.000514	0.004281
3638983	ALO1	5893.491301	9101.402763	-0.62697	6.83E-09	1.68E-07
3638984	CAALFM_CR09800CA	1204.547927	1612.63232	-0.42093	0.000708	0.005626
3638986	CAALFM_CR09830WA	1220.526826	513.6344142	1.2487	4.51E-05	0.000526
3638993	POP3	287.6664005	435.6413616	-0.59874	0.000407	0.003522
3638997	HMI1	635.0908673	824.6421759	-0.37681	0.008424	0.040161
3639009	CAALFM_CR09750CA	1708.092504	1236.458173	0.46617	0.000171	0.001677

3639012	IFA14	181.1477948	96.17721224	0.9134	0.0089	0.041943
3639026	DPB2	490.010963	343.6190636	0.512	0.006049	0.031001
3639029	CAALFM_CR09930WA	648.1338764	889.6135445	-0.45689	0.001003	0.007486
3639035	CAALFM_CR10060WA	2449.173629	3191.815305	-0.38208	0.000771	0.006018
3639037	ZCF39	3450.7351	4410.001495	-0.35388	0.001306	0.009269
3639044	MCD1	128.4413104	59.5805248	1.1082	0.00046	0.0039
3639045	DRS1	2085.89683	2580.096624	-0.30676	0.009546	0.044235
3639172	NA	2307.532422	976.2766026	1.241	5.61E-24	4.44E-22
3639174	NA	1971.059337	1309.706892	0.58973	1.29E-06	2.19E-05
3639177	NA	1563.40268	2266.051325	-0.53549	6.08E-06	9.09E-05
3639178	CAALFM_C603210CA	735.8789187	955.6724337	-0.37705	0.005414	0.028321
3639181	CAALFM_C603240WA	1104.732025	543.2406565	1.024	3.23E-05	0.000393
3639182	BMT4	328.9447384	629.2001117	-0.93567	1.45E-06	2.44E-05
3639184	AYR2	2229.233984	1271.876022	0.80959	1.78E-11	5.92E-10
3639190	CAALFM_C602970CA	817.9883318	503.3595652	0.70049	1.28E-06	2.17E-05
3639192	CAALFM_C602980CA	8863.270143	6915.609975	0.35798	0.001042	0.007686
3639198	CAALFM_C603320WA	1471.284533	795.4277145	0.88727	8.28E-12	2.88E-10
3639200	GLC3	7638.429716	4556.581515	0.74532	7.11E-12	2.50E-10
3639203	NA	146.400932	79.98268744	0.87217	0.001187	0.008548
3639204	NA	20.55487469	3.311639314	2.6339	0.002813	0.016891
3639205	CAALFM_C602560WA	2048.611396	321.5900642	2.6714	3.01E-09	7.90E-08
3639210	CDC37	5599.899216	3187.675243	0.8129	0.000148	0.001487

3639213	SAC6	13936.46955	9848.789714	0.50085	2.70E-06	4.30E-05
3639220	MIF2	2047.143041	1550.277724	0.40109	0.006811	0.033926
3639224	NA	602.2540223	431.4056146	0.48133	0.001628	0.01103
3639229	SAP6	193.798584	279.1835165	-0.52666	0.006201	0.03151
3639236	NA	8577.846338	7041.064857	0.28482	0.00766	0.037166
3639239	HPD1	1214.575281	193.4498082	2.6504	6.81E-66	2.23E-63
3639252	RAV2	840.2027317	1108.710571	-0.40007	0.002564	0.015798
3639254	POL5	865.9879948	1138.427503	-0.39462	0.002699	0.016363
3639257	CAALFM_C603150CA	596.1195475	770.8996205	-0.37094	0.009246	0.043168
3639260	MDR1	189.7405758	86.74738558	1.1291	3.79E-06	5.89E-05
3639268	SAP5	720.2895987	2599.601914	-1.8516	1.11E-49	2.70E-47
3639287	CAALFM_C100410CA	4230.448229	2715.228198	0.63974	1.12E-08	2.66E-07
3639294	HMX1	5752.686936	7968.743032	-0.47012	1.19E-05	0.000164
3639300	NA	327.3250954	189.0419115	0.79202	3.11E-05	0.000381
3639314	GCN20	1558.382082	2021.839406	-0.37562	0.001911	0.012638
3639326	VPS27	2898.030181	2261.448411	0.35783	0.002187	0.013983
3639327	SPE1	2566.550012	3294.108083	-0.36006	0.001531	0.010526
3639332	ASM3	420.7443288	624.7386177	-0.57031	0.000177	0.001717
3639335	NA	1481.341389	884.9254298	0.74328	5.28E-09	1.31E-07
3639341	CAALFM_C100160CA	7585.631266	9394.3573	-0.30853	0.003676	0.02086
3639344	CAALFM_C100830WA	2430.194236	1626.298767	0.57948	8.42E-07	1.50E-05
3639346	CAALFM_C100810WA	131.5835317	61.91009072	1.0877	0.000142	0.001433

3639347	NA	226.9024026	127.2996021	0.83384	0.000138	0.001406
3639349	HGC1	324.9101314	219.4079749	0.56642	0.006779	0.033845
3639355	CAALFM_C100880WA	374.7695498	179.3880852	1.0629	1.08E-06	1.89E-05
3639358	CAALFM_C100860WA	957.3927247	537.8243245	0.83198	3.85E-09	9.98E-08
3639365	CNS1	1035.417268	1341.839464	-0.374	0.00338	0.019605
3639366	CIS2	2771.820085	2236.943184	0.30931	0.011076	0.049834
3639378	RPO41	7509.060418	10321.41795	-0.45894	1.63E-05	0.000216
3639495	CAT1	104275.7931	54689.07704	0.93108	1.44E-18	8.85E-17
3639510	CAALFM_C106710WA	624.370037	824.3312277	-0.40082	0.003838	0.021595
3639516	NA	12375.55254	4548.736437	1.444	1.26E-39	1.80E-37
3639519	GLT1	52229.51067	26348.73771	0.98713	5.31E-21	3.74E-19
3639530	CAALFM_C106860WA	1995.700401	543.3249839	1.877	3.10E-46	6.16E-44
3639545	NA	91.3978097	49.16862475	0.89442	0.007027	0.03474
3639564	MEF2	684.2217081	1000.126582	-0.54765	4.72E-05	0.000546
3639570	ATC1	8656.121375	12290.33228	-0.50573	3.92E-05	0.000463
3639658	CAALFM_CR00660WA	28.21538777	61.79765407	-1.1311	0.00597	0.030646
3639667	MDH1	14009.21427	9993.347621	0.48734	5.15E-06	7.83E-05
3639674	NA	320.0030797	64.54798377	2.3096	7.90E-24	6.17E-22
3639681	MMS22	907.1445465	690.9722777	0.3927	0.004201	0.023244
3639682	CAALFM_CR00380WA	235.6204299	161.9303954	0.54109	0.010269	0.046876
3639685	LEU1	5026.528516	3783.384806	0.40988	0.000227	0.002139
3639688	ACC1	102397.2249	169507.7223	-0.72717	5.02E-10	1.40E-08

3639700	ALK2	1225.783256	400.7874424	1.6128	3.17E-30	3.35E-28
3639701	PCK1	29557.19179	55895.06476	-0.91921	1.59E-18	9.68E-17
3639704	CHT1	273.839771	148.3474018	0.88435	2.02E-05	0.000259
3639705	ZCF38	2753.59695	2089.980121	0.39783	0.000589	0.004803
3639707	POT1	20327.35466	7085.525994	1.5205	4.35E-45	7.92E-43
3639708	REP1	1365.742858	1053.89812	0.37395	0.00319	0.018669
3639712	CAALFM_CR00010CA	222.4508542	520.197106	-1.2256	0.000234	0.002189
3639713	PXA1	6262.570179	4333.446379	0.53124	1.16E-06	2.00E-05
3639715	CAALFM_CR00310CA	4934.675203	3497.509561	0.49663	9.18E-06	0.000131
3639717	CAALFM_CR00290WA	4266.73925	5802.761241	-0.44361	0.002361	0.014807
3639722	NA	3260.168063	2570.671165	0.3428	0.00227	0.014372
3639723	CAALFM_CR00490WA	912.3151117	1186.645658	-0.37929	0.003274	0.019088
3639724	MRPL6	720.8214061	1026.002169	-0.50932	0.000153	0.001529
3639726	CAALFM_CR00460CA	808.8616036	1050.588228	-0.37723	0.005174	0.027348
3639728	CAALFM_CR00420WA	1183.667575	894.2436935	0.40452	0.008895	0.041943
3639734	CAALFM_CR00090CA	3267.044955	2533.114398	0.36707	0.001259	0.009003
3639821	MSI3	65370.96984	29522.73017	1.1468	2.92E-06	4.61E-05
3639835	CAALFM_C301110CA	199.5677	106.9536578	0.89989	0.000111	0.001169
3639845	ATO2	11645.58729	966.9627073	3.5902	8.23E-37	1.06E-34
3639857	BMS1	2534.608194	3862.599809	-0.60781	8.38E-08	1.77E-06
3639859	NA	2072.140404	2941.41937	-0.50539	1.28E-05	0.000176
3639864	CAALFM_C105920WA	508.9015322	278.5668622	0.86936	1.95E-07	3.87E-06

3639875	NA	1492.018877	4111.317931	-1.4623	1.07E-35	1.30E-33
3639876	CAALFM_C105890WA	308.4765482	20.2624905	3.9283	1.00E-43	1.61E-41
3639877	NA	1767.053109	1322.199744	0.41841	0.000524	0.004338
3639889	PRC3	5723.554942	3816.213118	0.58477	1.07E-07	2.24E-06
3639894	UGA4	283.2464937	153.9042795	0.88002	1.44E-05	0.000195
3640077	CAALFM_CR01570WA	249.1285031	355.3213642	-0.51223	0.004449	0.024261
3640085	NA	61.44445278	120.5928696	-0.97279	0.000831	0.006387
3640089	PTR2	197.0508909	8.25098912	4.5779	7.16E-35	8.10E-33
3640100	PRS	763.657424	975.8242349	-0.3537	0.008523	0.040425
3640107	CAALFM_CR01950WA	2097.60365	2742.924765	-0.38697	0.000969	0.007273
3640112	NA	5363.318837	1950.106834	1.4596	3.30E-37	4.33E-35
3640134	LYS12	1700.61706	870.5852362	0.966	2.84E-14	1.24E-12
3640136	CAALFM_CR01410CA	429.282067	607.0595493	-0.49991	0.001246	0.008932
3640138	CAALFM_CR01430WA	1438.214496	2575.819662	-0.84075	0.000233	0.002186
3640149	HEM1	5208.652422	7607.230511	-0.54646	5.32E-07	9.84E-06
3640150	OPT1	1795.201754	489.4146472	1.875	8.67E-45	1.46E-42
3640157	CAALFM_CR01780WA	971.0156639	1224.391327	-0.3345	0.010059	0.046176
3640163	CAALFM_CR01810CA	162.9094991	101.3124525	0.68526	0.005341	0.027982
3640174	NA	813.178345	1070.036427	-0.39602	0.002599	0.015933
3640177	ZPR1	14157.7045	7268.658714	0.96183	6.22E-06	9.27E-05
3640179	NMA111	4188.017452	5420.430162	-0.37214	0.000777	0.006058
3640182	HMT1	1787.270807	2252.412987	-0.33371	0.005291	0.027813

3640193	CAALFM_C101620CA	952.6824431	447.655484	1.0896	2.59E-14	1.14E-12
3640194	CAALFM_C101630WA	3119.571349	1333.816843	1.2258	3.58E-25	3.02E-23
3640195	RPS42	2831.900987	3513.148892	-0.31099	0.004934	0.026381
3640198	NA	344.5093371	521.6016906	-0.59841	0.002931	0.017432
3640199	LSC1	3734.351733	2549.734929	0.55051	1.18E-06	2.03E-05
3640201	NA	4591.6268	2842.044703	0.69208	0.003467	0.01997
3640203	CAALFM_C101150CA	993.7453902	1386.770595	-0.48078	0.000173	0.001697
3640204	CAALFM_C101160CA	846.8256716	1079.187783	-0.34981	0.010959	0.04951
3640211	NA	6372.952982	637.1989921	3.3221	1.62E-85	8.86E-83
3640215	NA	857.4502567	653.0562069	0.39285	0.004831	0.025933
3640222	CAALFM_C101580WA	573.4483241	853.8829991	-0.57437	0.00017	0.00167
3640224	MRP20	1140.636927	1535.818105	-0.42917	0.000508	0.004237
3640225	NA	4397.321631	3105.678458	0.50172	8.86E-06	0.000127
3640226	CAALFM_C101490WA	17274.38663	13520.46502	0.35349	0.000732	0.005761
3640229	CAALFM_C101510WA	224.0587967	139.3392126	0.68528	0.001807	0.012044
3640235	TRM2	1535.035184	1945.360754	-0.34177	0.004746	0.025583
3640244	CAALFM_C603540WA	5117.505924	3245.353233	0.65707	4.46E-09	1.14E-07
3640247	POX1	6124.148817	2035.561198	1.5891	3.88E-28	3.69E-26
3640253	CAALFM_C603470WA	3018.778517	2172.599828	0.47454	3.55E-05	0.000425
3640257	SAP4	438.5856243	266.8926708	0.7166	2.91E-05	0.00036
3640269	NA	6552.445713	4865.269321	0.42951	6.72E-05	0.000744
3640273	CAALFM_C603430CA	3598.011118	4387.665043	-0.28625	0.010118	0.046351

3640274	NA	1634.919149	2140.657904	-0.38883	0.001099	0.008048
3640281	ALS9	1271.556485	1850.112018	-0.54102	9.25E-06	0.000132
3640286	CAALFM_C603810WA	886.5965921	468.6725536	0.9197	7.74E-07	1.38E-05
3640290	SNQ2	11835.47073	6934.082216	0.77134	5.91E-13	2.34E-11
3640293	CAALFM_C603880WA	726.1783611	217.9770286	1.7361	1.78E-09	4.75E-08
3640295	CAALFM_C603600CA	8288.947415	114.9516644	6.1721	#######	#######
3640297	CAALFM_C603620CA	1410.957418	972.5415784	0.53684	2.63E-05	0.000328
3640303	CAALFM_C603730CA	1079.794485	1366.368432	-0.33959	0.007147	0.03528
3640306	SHM2	13991.20652	9435.983781	0.56828	1.14E-07	2.38E-06
3640307	ORM1	1530.419327	919.473643	0.73505	0.00078	0.006078
3640308	CAALFM_C603780CA	4027.682764	3171.774193	0.34466	0.002429	0.015135
3640309	HGT10	21546.24004	4565.585335	2.2386	7.37E-13	2.90E-11
3640392	ATO9	61.44445278	7.633461184	3.0089	1.23E-08	2.90E-07
3640394	ATO10	118.1387418	26.21202287	2.1722	3.07E-10	8.86E-09
3640400	NA	1973.692332	1420.051275	0.47495	0.00036	0.0032
3640402	CAALFM_CR01260WA	1013.646671	562.2935792	0.85016	1.16E-07	2.41E-06
3640405	CDC27	940.5153388	724.4523426	0.37656	0.005684	0.029499
3640406	CAALFM_CR01220WA	2142.383968	434.099726	2.3031	1.46E-15	7.11E-14
3640407	HSE1	2252.771552	1754.635787	0.36053	0.002563	0.015798
3640408	OYE22	140.5767731	56.63343094	1.3116	0.001446	0.01006
3640416	PWP2	2180.912305	2709.018694	-0.31284	0.007287	0.035779
3640422	SLN1	6681.739027	8415.64219	-0.33285	0.001809	0.012045

3640435	GSY1	24370.36765	16151.97813	0.59342	1.75E-06	2.91E-05
3640511	ERG4	1790.013387	1212.997353	0.56139	5.33E-06	8.06E-05
3640519	DOT5	12308.65389	8735.363793	0.49473	0.000472	0.003981
3640522	NA	15730.54837	32022.28401	-1.0255	1.94E-21	1.40E-19
3640527	CAALFM_C300270CA	852.1730659	1117.29887	-0.3908	0.003187	0.018665
3640530	CAALFM_C300730WA	1142.583367	881.472371	0.37431	0.004677	0.025271
3640531	PMA1	22584.7412	34159.36328	-0.59693	2.82E-06	4.47E-05
3640547	UTP8	2297.518088	3099.17111	-0.43181	0.000201	0.001931
3640553	CAALFM_C300420WA	2398.396284	2989.268611	-0.31772	0.005902	0.030365
3640557	CAALFM_C300230CA	470.9890469	204.0558514	1.2067	8.85E-12	3.06E-10
3640558	HGT19	42214.83315	13979.45636	1.5944	2.43E-50	6.12E-48
3640560	CAALFM_C300210CA	2384.362504	565.5806041	2.0758	4.03E-25	3.34E-23
3640561	ETR1	5251.817877	2563.489198	1.0347	1.00E-10	3.02E-09
3640564	DAL1	711.7579612	332.8153826	1.0967	2.82E-07	5.45E-06
3640573	NGT1	251.350157	101.8728884	1.3029	7.18E-09	1.74E-07
3640585	SOF1	2805.577497	3545.39128	-0.33765	0.002968	0.01757
3640710	NA	3299.001115	2535.394735	0.37982	0.000723	0.005719
3640722	MRPL3	1542.474206	1988.746754	-0.36661	0.002056	0.013405
3640723	ERD1	232.4114653	152.8949707	0.60414	0.0043	0.023626
3640742	GCS1	14875.08211	19950.59521	-0.42353	0.002669	0.016258
3640745	CAT8	2022.642884	1461.193783	0.46909	9.47E-05	0.001012
3640746	NTG1	972.2734083	1325.814468	-0.44744	0.000436	0.003731

3640748	MLT1	8610.865612	7119.987259	0.27428	0.010276	0.046876
3640750	PLB5	3751.502193	2808.207082	0.41782	0.000201	0.001931
3640751	ADH2	3879.302931	545.26539	2.8308	#######	#######
3640756	SDS24	5346.102954	6947.625591	-0.37803	0.002308	0.014557
3640762	DIP2	1758.611616	2673.318005	-0.60419	3.09E-05	0.00038
3640767	TKL1	31360.25856	22137.83407	0.50242	0.000425	0.003655
3640939	YDJ1	10357.7338	5322.56188	0.96052	3.29E-07	6.24E-06
3640941	QDR1	271.4131064	686.4545654	-1.3387	2.27E-17	1.23E-15
3640943	NA	21.62062938	5.528768578	1.9674	0.010967	0.049514
3640948	CAALFM_CR04580WA	1270.439147	1762.324593	-0.47215	0.000101	0.001073
3640950	CAALFM_CR04600WA	1752.714614	2319.345881	-0.40413	0.000575	0.004707
3640956	TPO4	2489.550841	1057.800052	1.2348	0.002639	0.016121
3640968	NA	1041.641888	1523.556242	-0.54858	1.23E-05	0.000169
3640973	EAF7	542.9493193	708.8481103	-0.38466	0.008695	0.041152
3640974	CAALFM_CR04140WA	1796.022616	2342.891028	-0.38348	0.001045	0.0077
3640976	CAALFM_CR04160CA	1528.833966	2144.116205	-0.48795	5.37E-05	0.000612
3640977	CAALFM_CR04170WA	2749.481759	3505.658597	-0.35053	0.002272	0.014372
3640983	RBR2	1517.02744	1042.815095	0.54076	1.76E-05	0.00023
3640986	NA	2117.305521	344.6863382	2.6189	4.20E-34	4.67E-32
3640989	CAALFM_CR04240CA	5176.093431	6919.825476	-0.41887	0.000138	0.001406
3640995	CAALFM_CR04300WA	3622.634881	4502.361977	-0.31364	0.005398	0.028263
3641004	NA	496.9394385	693.1876596	-0.48018	0.001095	0.008032

3641012	LAP3	5784.590694	3800.779288	0.60592	3.55E-08	7.89E-07
3641016	FUM11	641.5625192	410.1329415	0.6455	2.43E-05	0.000304
3641026	CAALFM_CR04110WA	491.5838037	743.4797772	-0.59686	5.09E-05	0.000584
3641104	RCE1	1065.405727	1403.13028	-0.39725	0.001575	0.010796
3641117	MNN22	1467.726691	3063.628592	-1.0617	1.76E-06	2.91E-05
3641126	SAP10	1487.687795	1084.597125	0.45591	0.001332	0.009413
3641148	CAALFM_C405010WA	2184.90011	2989.921238	-0.45254	0.000104	0.001099
3641157	SCS7	4989.473315	7377.505448	-0.56425	1.93E-07	3.85E-06
3641165	CAALFM_C404820CA	417.161765	618.5361029	-0.56825	0.000183	0.001775
3641180	LYS4	1832.515688	1359.045046	0.43123	0.000392	0.003427
3641186	PDB1	9623.676261	7712.781332	0.31934	0.00278	0.016761
3641196	CAALFM_C210470CA	1628.319611	1134.245353	0.52165	3.05E-05	0.000375
3641197	MDH1-3	5481.864641	3103.692317	0.82068	1.25E-13	5.27E-12
3641212	NA	754.694504	1030.020032	-0.44871	0.006315	0.031889
3641214	JEN2	15166.13303	4512.145714	1.749	3.20E-15	1.50E-13
3641215	NA	246.7850629	140.3221597	0.81451	0.006231	0.031563
3641218	CAALFM_C404010WA	1341.168537	870.9761434	0.62279	1.49E-06	2.50E-05
3641221	NA	462.4595493	731.6659137	-0.66186	6.99E-06	0.000103
3641243	CAALFM_C210810WA	634.327188	922.5551669	-0.54041	0.000887	0.006752
3641258	SGS1	884.4430017	644.8342006	0.45584	0.000934	0.007057
3641344	IFA21	544.773973	390.0101232	0.48215	0.001943	0.012794
3641346	CAALFM_C703150WA	305.354268	713.5089895	-1.2244	3.13E-15	1.48E-13

3641349	NA	1214.276666	1641.931109	-0.4353	0.000436	0.003731
3641353	NA	11022.76551	8683.595146	0.34412	0.001508	0.01041
3641380	CAALFM_C703030WA	2152.080565	1341.365103	0.68203	3.66E-08	8.12E-07
3641389	IFM1	1040.537571	1345.905683	-0.37125	0.003536	0.02024
3641391	NA	575.7415019	205.1784706	1.4885	3.03E-18	1.79E-16
3641400	ERG5	6177.530976	4112.035664	0.58718	7.71E-08	1.64E-06
3641536	NA	179.1662532	4.939349806	5.1808	2.63E-35	3.08E-33
3641539	NA	103.4431278	54.19230205	0.93268	0.002515	0.015615
3641540	NAM7	4980.484854	6187.195114	-0.313	0.003746	0.021147
3641542	NA	4796.558876	7028.052934	-0.55113	3.18E-07	6.05E-06
3641545	IFG3	2170.696421	2879.729784	-0.40778	0.002654	0.016193
3641547	MET14	3581.733102	4917.386938	-0.45723	0.002008	0.013143
3641548	MRP17	468.6924091	631.6421143	-0.43047	0.003745	0.021147
3641554	CAALFM_C500350CA	1217.08731	1698.676976	-0.48098	8.04E-05	0.000873
3641562	NA	457.095674	605.9369301	-0.40667	0.007079	0.03497
3641570	HMS1	2317.963697	3041.31136	-0.39184	0.000632	0.005087
3641571	ERG11	8358.944266	6675.875428	0.32436	0.002542	0.015729
3641572	THR1	497.9536105	371.9085436	0.42106	0.0092	0.043109
3641587	PET9	42554.41695	31693.63619	0.42512	5.00E-05	0.000576
3641593	FAS1	88980.99715	140391.8019	-0.65789	3.44E-10	9.89E-09
3641597	NA	1080.703352	741.882797	0.54271	4.36E-05	0.00051
3641598	CAALFM_C601890CA	1892.301936	2453.995231	-0.37499	0.001396	0.009797

3641600	FRP5	44.74735604	10.55244588	2.0842	0.00011	0.001163
3641601	FRP6	3587.452456	2147.257442	0.74046	1.09E-06	1.89E-05
3641602	CAALFM_C601420CA	5295.195582	3695.486692	0.51892	2.60E-06	4.17E-05
3641611	PTK2	6876.330792	8332.162457	-0.27705	0.010231	0.046736
3641614	CAALFM_C601680CA	177.7385999	69.57078739	1.3532	2.48E-07	4.84E-06
3641615	MAE1	2278.918714	3140.20992	-0.46251	5.56E-05	0.000629
3641619	TRY5	1770.588871	674.8910632	1.3915	5.08E-27	4.56E-25
3641620	CAALFM_C601490CA	294.0210465	112.5096618	1.3859	8.92E-11	2.71E-09
3641625	CAALFM_C601870CA	2523.328695	2048.695319	0.30062	0.009986	0.046002
3641639	NA	1956.054727	2431.606055	-0.31396	0.007865	0.037964
3641644	TRA1	14408.6155	18424.58253	-0.3547	0.000831	0.006387
3641646	TIF3	2128.443292	2633.747862	-0.30732	0.007025	0.03474
3641651	NA	602.132236	773.7342777	-0.36176	0.011013	0.049652
3641652	CAALFM_C601560WA	738.9262151	556.0954328	0.4101	0.004402	0.024042
3641716	KIP3	796.2411359	586.2629847	0.44166	0.002152	0.013813
3641720	NA	2753.093324	2193.59403	0.32776	0.004581	0.024856
3641724	CAALFM_C305680WA	892.1654779	693.8622795	0.36266	0.00895	0.042115
3641731	FAA2	3075.834704	1656.861595	0.89252	2.03E-14	9.07E-13
3641740	ZCF35	3401.973079	2705.957416	0.33023	0.004149	0.02302
3641741	MRR1	3840.236686	2301.495536	0.73862	1.11E-10	3.31E-09
3641743	CTA4	8733.511265	12724.24079	-0.54295	2.60E-07	5.03E-06
3641745	CAALFM_C306040WA	564.6050505	272.9528925	1.0486	1.85E-10	5.45E-09

3641746	CAALFM_C306050CA	698.6854503	1062.964275	-0.60538	6.60E-06	9.80E-05
3641747	ELP3	3647.282866	4601.226311	-0.3352	0.002692	0.016338
3641752	DED1	17995.0063	23682.96842	-0.39625	0.000136	0.001397
3641755	NA	453.332821	713.6512827	-0.65465	0.004401	0.024042
3641758	CAALFM_C305850WA	645.5503238	473.8675069	0.44605	0.002739	0.01656
3641759	CAALFM_C305860CA	529.339401	688.2210743	-0.37868	0.011119	0.049995
3641762	GDA1	2276.213375	2867.408208	-0.33311	0.005442	0.028421
3641774	TUB4	128.724765	75.99817555	0.76025	0.005742	0.029729
3641775	NA	400.2093952	20.40303633	4.2939	4.45E-46	8.58E-44
3641776	NA	16813.81615	20993.87111	-0.32032	0.002211	0.014093
3641785	NA	633.3082356	468.8710651	0.43372	0.004202	0.023244
3641794	GAT1	1729.906443	851.7194671	1.0222	1.67E-13	6.97E-12
3641796	NA	3878.059847	2681.542632	0.53227	0.001537	0.010559
3641805	NA	282.8295523	179.471539	0.65618	0.000869	0.006647
3641817	PHO4	1068.115846	823.4334818	0.37534	0.004571	0.024856
3641874	NIT2	1233.769246	692.5991145	0.83298	4.43E-10	1.25E-08
3641876	NA	12487.81932	7726.358209	0.69266	3.61E-10	1.03E-08
3641877	PEX13	2308.86037	1344.589794	0.78001	9.61E-11	2.91E-09
3641879	CAALFM_CR09140CA	22003.16748	15945.35268	0.46458	0.001027	0.007642
3641881	ERG13	4044.717539	2397.45137	0.75454	2.91E-11	9.60E-10
3641892	CAALFM_CR09010CA	5843.678685	3991.25564	0.55003	5.31E-07	9.84E-06
3641911	CAALFM_CR08920WA	19402.31509	4716.414207	2.0405	1.24E-76	5.42E-74

3641917	LIP7	183.255903	260.6892823	-0.50847	0.010873	0.049191
3641919	NA	2786.524759	1840.993139	0.59798	9.26E-06	0.000132
3641922	SCO1	2297.790662	3191.081845	-0.4738	3.27E-05	0.000397
3641926	NA	738.2457601	570.2125005	0.3726	0.009475	0.04405
3641928	NA	1125.948233	683.9299827	0.71922	0.001032	0.00766
3641929	CAALFM_CR09110CA	1035.389087	1661.543594	-0.68235	7.40E-07	1.33E-05
3641930	TUB1	4685.327849	3744.882811	0.32323	0.003458	0.019931
3642006	CAALFM_C111890WA	2365.838114	1871.102725	0.33846	0.00412	0.022889
3642010	CAALFM_C111850WA	4862.414995	3370.067514	0.5289	0.004867	0.026064
3642011	IRA2	13700.08991	18039.71916	-0.39699	0.000175	0.00171
3642016	NA	825.3385291	1140.310817	-0.46637	0.000468	0.003958
3642023	CAALFM_C112240CA	1282.951669	1686.554786	-0.39461	0.001151	0.008317
3642025	CAALFM_C112070CA	569.1268024	370.0278506	0.62112	9.10E-05	0.000978
3642026	NA	306.5933917	200.8838842	0.60996	0.001408	0.009855
3642030	SER33	4537.155986	6563.789151	-0.53274	8.87E-07	1.57E-05
3642032	KIP4	1155.667579	1827.747456	-0.66134	8.68E-08	1.83E-06
3642039	NA	929.2454006	1223.829143	-0.39727	0.002327	0.014635
3642055	PXA2	4902.847431	3494.095095	0.4887	8.67E-06	0.000125
3642062	RIB3	2279.893003	3655.552003	-0.68112	1.92E-09	5.11E-08
3642073	CAALFM_CR08170CA	770.7007655	999.5116751	-0.37505	0.005606	0.029141
3642090	ACO1	50476.4032	36389.77872	0.47208	3.69E-05	0.000441
3642097	HSP104	114347.6092	51172.94372	1.16	0.000841	0.00645

3642101	NA	1876.383179	624.6911363	1.5867	1.48E-05	0.000198
3642102	CAALFM_CR08280WA	1544.521171	789.0020737	0.96906	8.30E-06	0.00012
3642108	PEX19	4635.023808	2856.166139	0.6985	3.48E-10	9.97E-09
3642109	CAALFM_CR08610WA	340.6246978	236.3026078	0.52755	0.003531	0.020229
3642115	CAALFM_CR08400CA	438.6723087	611.2970437	-0.47873	0.001655	0.011182
3642117	CAALFM_CR08420WA	8213.135103	3397.547071	1.2734	0.000115	0.001203
3642126	CAALFM_CR08670CA	7647.998927	1296.687829	2.5603	#######	#######
3642127	NA	1589.636526	1234.43606	0.36484	0.003239	0.018906
3642128	ECI1	14630.44572	3166.446557	2.208	1.18E-86	7.02E-84
3642135	TSR1	2108.925171	2786.165851	-0.40177	0.000562	0.004612
3642136	NA	1686.513077	2299.731648	-0.44742	0.000175	0.00171
3642237	NA	2865.015687	2068.320035	0.47008	4.10E-05	0.000482
3642244	CYM1	4780.236699	5974.017763	-0.32162	0.00292	0.0174
3642247	CAALFM_C306700CA	4783.072064	5906.993552	-0.30449	0.004685	0.025295
3642249	FUN31	4621.958218	3238.621012	0.51312	4.06E-06	6.24E-05
3642250	NA	151.6863632	96.79386649	0.6481	0.010081	0.046244
3642277	NA	670.2581317	514.2247066	0.38232	0.008481	0.040323
3642278	JEN1	1694.512087	69.23522478	4.6132	4.58E-92	3.00E-89
3642279	LYS9	5653.419323	4163.429281	0.44135	5.55E-05	0.000629
3642282	GLG2	1234.654711	874.5969836	0.49742	0.000125	0.001289
3642294	CAALFM_C207920WA	477.8005165	671.9457167	-0.49194	0.001109	0.008065
3642295	GDH2	70612.93478	101280.7674	-0.52036	4.82E-07	9.01E-06

3642296	PRS5	3287.758038	4273.862359	-0.37844	0.00075	0.005884
3642303	YVC1	1206.737939	802.1089589	0.58924	6.72E-06	9.95E-05
3642307	SIT1	4322.364325	13365.58159	-1.6286	7.08E-40	1.06E-37
3642318	CAALFM_C208170WA	737.1999465	545.4314239	0.43466	0.002524	0.015649
3642323	NA	2271.81951	1688.204489	0.42836	0.006093	0.031178
3642325	NA	556.5193555	830.0022895	-0.57668	4.08E-05	0.00048
3642329	PHM7	22472.14945	17343.79937	0.37372	0.000373	0.003296
3642331	ARA1	6492.612162	5031.596255	0.36778	0.000787	0.006107
3642335	IFM3	1268.527809	475.9440903	1.4143	1.63E-24	1.32E-22
3642336	CAALFM_C208090WA	114.379349	178.5729194	-0.64269	0.007295	0.035792
3642339	NA	805.7263026	1056.031796	-0.39029	0.002985	0.017658
3642341	PDS5	1198.302046	909.5378514	0.39779	0.00241	0.015053
3642345	NA	85.1649499	41.59138189	1.034	0.002848	0.017013
3642465	NA	336.1532083	458.29051	-0.44714	0.006227	0.031563
3642467	CUE5	4825.896382	3768.678319	0.35674	0.001306	0.009269
3642468	NA	14080.00922	8012.012076	0.81341	0.006377	0.03218
3642482	CAALFM_C105360CA	2751.143424	3485.614864	-0.34138	0.002516	0.015615
3642483	YPT53	727.9748332	1267.411034	-0.79992	1.40E-09	3.77E-08
3642484	ZCF2	4686.085428	6176.83419	-0.39848	0.00022	0.002098
3642487	MTM1	1200.803694	1613.329807	-0.42604	0.000518	0.004298
3642489	CAALFM_C105440CA	11775.88321	7748.558485	0.60384	1.51E-05	0.000201
3642493	CAALFM_C105420WA	750.7114797	967.7700099	-0.36641	0.006195	0.03151

3642494	TRP99	4503.991524	3215.024889	0.48637	1.27E-05	0.000174
3642503	CAALFM_C105540CA	1229.891527	1607.35566	-0.38616	0.001602	0.010926
3642505	NA	190.4561325	293.1600384	-0.62223	0.001288	0.00918
3642506	RSM22	1209.031117	1650.288419	-0.44887	0.007215	0.035508
3642570	RFG1	2931.294347	2013.358302	0.54193	1.09E-05	0.000153
3642573	CAALFM_CR02680WA	284.1939223	183.0063043	0.63498	0.001472	0.010196
3642577	RPC31	184.8053426	263.2428479	-0.51039	0.010444	0.047511
3642582	CAALFM_CR02780WA	421.7901425	292.1217466	0.52996	0.001939	0.012782
3642584	NA	600.5112728	340.0842984	0.8203	5.61E-05	0.000632
3642585	CAALFM_CR02890CA	1251.226562	1589.816264	-0.34552	0.005768	0.029839
3642590	CAALFM_CR02950CA	1014.145517	1502.141276	-0.56676	5.42E-06	8.16E-05
3642605	NA	1300.405525	1772.483511	-0.44681	0.000268	0.002452
3642612	CFL5	14.597229	45.21134833	-1.631	0.001104	0.008052
3642616	GPR1	6012.574331	4642.645543	0.37304	0.010913	0.049336
3642621	CYC3	3083.432754	3981.842565	-0.3689	0.001033	0.00766
3642624	NA	2766.493451	1934.668636	0.51597	1.15E-05	0.00016
3642627	NA	1637.507482	3159.647634	-0.94826	6.56E-16	3.31E-14
3642631	TRX2	341.8239393	155.5890821	1.1355	6.22E-09	1.53E-07
3642636	NA	321.7878512	443.8097706	-0.46383	0.004872	0.026071
3642639	CFL4	83.24191121	165.0452705	-0.98748	0.002038	0.013314
3642648	CAALFM_C501050CA	381.1090351	532.5485384	-0.48271	0.002178	0.01394
3642661	PUS4	1691.463471	2110.884754	-0.31958	0.007281	0.035776

3642662	NA	467.9534512	639.3335412	-0.4502	0.002835	0.01697
3642663	CAALFM_C501140CA	1820.513712	2341.599754	-0.36315	0.001845	0.012265
3642779	CBF1	554.521333	940.6040229	-0.76234	3.44E-08	7.68E-07
3642780	PDC11	15937.98455	34610.46119	-1.1187	3.38E-15	1.57E-13
3642785	XUT1	3003.268961	1605.721834	0.90331	1.59E-14	7.19E-13
3642786	CSO99	3059.163149	1744.390795	0.81041	4.53E-12	1.62E-10
3642790	NA	889.7587544	667.6502567	0.41432	0.002943	0.017482
3642793	CAALFM_C406440CA	634.5755408	484.6140959	0.38896	0.00846	0.040274
3642794	CAALFM_C406430CA	2053.713578	1560.739727	0.39601	0.000852	0.00653
3642817	MPH1	748.7849809	517.618926	0.53266	0.000236	0.002203
3642818	CAALFM_C406170CA	1530.115112	1907.813597	-0.31828	0.008489	0.040324
3642821	CAALFM_C406140CA	362.4771991	252.0746214	0.52404	0.003222	0.018839
3642902	PDA1	3568.307434	2752.783218	0.37435	0.002116	0.013651
3642918	CAALFM_C406790WA	901.7338689	1188.160058	-0.39796	0.002396	0.014981
3642920	CAALFM_C406770WA	2131.527011	1710.350294	0.3176	0.007944	0.038289
3642933	CAALFM_C406910WA	109.8294155	213.317023	-0.95773	3.03E-05	0.000374
3642939	MSH2	1609.58593	1196.245013	0.42818	0.000497	0.004163
3642947	NOP1	1201.609396	1563.46719	-0.37978	0.002136	0.013743
3642964	MET16	1899.259413	3525.747191	-0.89249	5.93E-15	2.74E-13
3642965	NA	306.8218034	1784.471271	-2.54	3.70E-73	1.52E-70
3642966	CAALFM_C407010CA	718.7414794	1142.72471	-0.66893	6.32E-07	1.14E-05
3642970	ZRT1	85.05486425	147.8712934	-0.79788	0.002191	0.013997

3642975	UGA6	376.3506309	224.2041578	0.74726	0.001105	0.008052
3642978	SGT2	8731.07604	6797.777046	0.3611	0.00077	0.006018
3642985	NA	734.0893669	385.4625543	0.92936	8.44E-10	2.31E-08
3642987	TOM1	26919.80248	32978.00769	-0.29284	0.004957	0.026428
3642989	RNR1	2025.88431	1431.815909	0.50071	0.000113	0.001178
3642993	CAALFM_C203130WA	1086.464228	1437.483476	-0.4039	0.001379	0.009705
3642994	AMO1	2000.423704	1084.151747	0.88374	7.66E-13	2.99E-11
3643011	NA	1828.674391	2271.833076	-0.31306	0.007939	0.038289
3643012	CAALFM_C202950WA	1741.180342	428.3048696	2.0234	0.000502	0.004199
3643015	CAALFM_C202930CA	1917.90905	3137.002854	-0.70985	8.50E-10	2.31E-08
3643026	ALD5	166871.7082	76205.32858	1.1308	3.44E-11	1.10E-09
3643029	CAALFM_C202790CA	470.3753354	249.2101077	0.91645	1.19E-07	2.47E-06
3643032	LHS1	3479.628803	2709.135499	0.3611	0.001417	0.009889
3643101	UME1	1865.706191	2327.20771	-0.31888	0.006963	0.03455
3643115	CAALFM_C703990CA	558.1871212	748.8117817	-0.42385	0.003095	0.018207
3643119	CAALFM_C703780CA	1027.26829	630.6336791	0.70394	3.18E-07	6.05E-06
3643123	UTP18	1851.662031	2729.926822	-0.56004	0.000376	0.003308
3643125	FAA2-3	22033.75914	3262.03435	2.7559	#######	#######
3643127	CAALFM_C704160WA	373.4801637	506.3373893	-0.43907	0.006156	0.031417
3643129	CAALFM_C704140CA	458.2364126	642.7549961	-0.48818	0.002755	0.016639
3643144	CAALFM_C703860WA	23362.45749	16220.01981	0.52642	5.77E-07	1.05E-05
3643147	NA	300.7258905	162.1552687	0.89107	8.58E-06	0.000124

3643148	NA	771.6165524	482.5085296	0.67733	3.95E-06	6.11E-05
3643149	SLA2	5391.893984	4301.338421	0.32601	0.003039	0.01791
3643150	CAALFM_C703800WA	171.7575531	110.9100605	0.63098	0.008418	0.040161
3643165	CAALFM_C704240CA	1600.278912	2615.848289	-0.70896	2.42E-09	6.41E-08
3643166	NRG1	1854.1818	1332.522074	0.47662	0.000944	0.007117
3643179	NA	297.7253966	199.0602832	0.58078	0.002731	0.016529
3643180	KRE1	1649.126298	995.1318875	0.72874	7.11E-09	1.73E-07
3643191	SGD1	1377.953304	1927.737904	-0.48438	6.83E-05	0.000753
3643199	NA	301.9485332	53.85586576	2.4871	5.02E-16	2.57E-14
3643200	DEM1	367.5975018	270.1472181	0.44438	0.011022	0.049659
3643201	CIT1	62970.51898	19686.82992	1.6774	1.20E-55	3.15E-53
3643204	CAALFM_CR03530WA	9718.542269	8009.981226	0.27894	0.008378	0.04
3643216	NCS2	2158.921537	2885.34288	-0.41843	0.000283	0.002584
3643218	YVH1	819.8060652	1220.039648	-0.57357	3.44E-05	0.000415
3643221	SMC1	770.2005997	588.9289869	0.38714	0.00737	0.036107
3643222	CAALFM_CR03690WA	4495.803165	1951.060798	1.2043	5.35E-26	4.68E-24
3643285	CAALFM_C114380CA	411.6878041	577.7598868	-0.48892	0.001524	0.010499
3643318	FTR1	4637.104054	8086.342351	-0.80226	1.86E-05	0.00024
3643320	SET3	2337.5105	1538.794181	0.60317	3.40E-07	6.43E-06
3643321	NA	711.0211432	939.8740583	-0.40257	0.002777	0.016757
3643327	CAALFM_C114470WA	231.6876681	324.3122847	-0.4852	0.008049	0.038712
3643339	CAALFM_C114020WA	355.3168518	60.16994357	2.562	4.24E-10	1.20E-08

3643343	CAALFM_C114050CA	3494.38292	4448.481497	-0.34828	0.001735	0.01166
3643423	NA	672.8802461	982.2226402	-0.5457	5.07E-05	0.000583
3643427	RPC40	1405.976702	1952.797451	-0.47397	0.000103	0.001091
3643430	BIO32	1185.558972	587.636839	1.0126	1.67E-08	3.85E-07
3643436	PEX6	9505.550039	3955.765846	1.2648	1.70E-30	1.82E-28
3643437	NA	241.9833164	337.4736408	-0.47987	0.00939	0.043749
3643440	ITR1	8995.54583	6395.020063	0.49226	0.004586	0.024856
3643442	NA	864.8589568	1136.773431	-0.39441	0.002611	0.015979
3643446	NA	588.2822054	870.1899605	-0.56482	5.55E-05	0.000629
3643451	CAALFM_C205060CA	526.6678438	738.5395537	-0.48778	0.00135	0.009526
3643454	MAK5	1908.208492	2470.809905	-0.37277	0.001523	0.010499
3643458	CAALFM_C205130WA	1267.333348	922.7536784	0.45778	0.000394	0.003433
3643461	CAALFM_C205160CA	3846.670277	4856.807589	-0.3364	0.00257	0.015798
3643467	NA	1453.397755	1960.290366	-0.43164	0.000376	0.003308
3643468	AAT1	5424.301686	2209.452119	1.2957	2.28E-20	1.56E-18
3643475	NA	192.2491445	559.573106	-1.5413	3.16E-18	1.85E-16
3643482	TPK2	1993.585373	1398.055626	0.51194	2.02E-05	0.000259
3643487	CAALFM_C207250CA	121.3828083	184.7192159	-0.60577	0.008744	0.041297
3643495	LTE1	517.3408853	307.051359	0.75264	4.44E-06	6.81E-05
3643497	NA	397.6609444	546.468842	-0.4586	0.003617	0.020614
3643498	CAALFM_C207030CA	502.8654425	708.0346919	-0.49365	0.00073	0.00576
3643500	CAALFM_C207060WA	665.7948827	861.071082	-0.37105	0.008121	0.038945

3643501	CAALFM_C207070WA	4294.293305	2427.849911	0.82274	3.51E-13	1.40E-11
3643502	CAALFM_C207080CA	940.3935526	1287.70076	-0.45346	0.000409	0.003532
3643503	OLE2	1953.754629	2516.078346	-0.36493	0.001981	0.013009
3643513	CAALFM_C207190CA	1103.596887	1585.634988	-0.52285	2.35E-05	0.000295
3643515	CAALFM_C206770WA	170.1764719	108.8615862	0.64454	0.008142	0.038984
3643516	CAALFM_C206780CA	1620.977654	1275.82893	0.34543	0.005585	0.02908
3643518	CAALFM_C206800CA	687.0770146	446.978243	0.62027	3.82E-05	0.000453
3643521	PRB1	12542.05234	9359.164324	0.42232	8.34E-05	0.000904
3643531	NA	201.1721824	84.9510201	1.2437	2.97E-07	5.69E-06
3643648	CAALFM_C305320WA	3800.844965	5763.055794	-0.60051	3.90E-08	8.56E-07
3643651	MNN14	1337.462866	1750.962223	-0.38865	0.00122	0.008763
3643652	ATO5	73.51317204	141.0521243	-0.94015	0.000557	0.004579
3643653	GTT1	46.91396719	4.911240642	3.2559	3.15E-07	6.03E-06
3643656	NA	21.74241562	3.844839757	2.4995	0.003446	0.019899
3643662	GAP2	42569.87447	14668.17964	1.5371	1.00E-46	2.12E-44
3643663	BAT22	7807.270819	5513.829809	0.50176	3.47E-06	5.41E-05
3643676	CAALFM_C305360CA	10483.51758	6915.157607	0.60029	2.56E-08	5.82E-07
3643679	CAALFM_C305380WA	3091.045645	1983.725698	0.63988	2.83E-08	6.38E-07
3643734	CCT7	2657.192383	2154.723995	0.3024	0.009397	0.043751
3643735	CCN1	342.2725224	237.0606815	0.52989	0.005283	0.02779
3643736	DAL52	87.41824552	44.2573841	0.98202	0.003938	0.022097
3643746	CAALFM_C502070CA	1070.097388	1407.510067	-0.3954	0.001772	0.011873

3643758	STI1	24549.14842	14595.60478	0.75014	0.001307	0.009269
3643760	FCR3	2800.688566	2039.743348	0.45739	0.007509	0.036544
3643764	NA	5499.98807	4128.921408	0.41366	0.000139	0.001406
3643769	CAALFM_C502040WA	2357.488905	2986.968902	-0.34143	0.002686	0.016324
3643773	ACH1	32531.77502	23057.24044	0.49663	1.01E-05	0.000141
3643780	CAALFM_C209870WA	1593.674593	1272.407476	0.3248	0.009009	0.042338
3643784	IPK2	639.2658813	375.4169471	0.76792	7.38E-07	1.33E-05
3643793	NA	1105.234331	1469.220773	-0.4107	0.001124	0.008151
3643795	PMI1	499.0427663	370.5057064	0.42967	0.007973	0.038403
3643797	CAALFM_C209620WA	1179.451359	902.3796249	0.38631	0.002838	0.016974
3643800	NA	745.9496154	577.4251978	0.36944	0.009515	0.044206
3643808	LEU42	5973.300935	2889.313262	1.0478	6.03E-10	1.67E-08
3643811	NA	1284.32082	888.0921549	0.53222	4.77E-05	0.00055
3643816	CAALFM_C209510CA	3877.176202	4795.066153	-0.30654	0.005528	0.028825
3643821	NA	396.2449917	260.0717544	0.60748	0.00057	0.004671
3643873	SPO11	204.7664468	305.1434306	-0.57551	0.002584	0.015859
3643875	APE3	2269.365984	1670.306663	0.44218	0.002419	0.015096
3643876	JEM1	307.1238788	218.6780103	0.49001	0.009572	0.044313
3643886	NA	789.0725481	368.3439218	1.0991	3.32E-13	1.34E-11
3643887	CAALFM_C208700CA	3221.260846	1848.372744	0.80137	6.43E-12	2.27E-10
3643888	CAALFM_C208690CA	768.5362942	385.4353188	0.99562	0.000241	0.002247
3643903	NA	272.1334435	144.6431079	0.91182	9.99E-06	0.000141

3643906	UTP15	2356.649422	3011.519863	-0.35375	0.002093	0.013543
3643907	CAALFM_C208660CA	1298.922829	675.0008788	0.94435	1.25E-12	4.78E-11
3643912	NA	1192.841106	922.9802991	0.37003	0.004097	0.022812
3643922	NA	763.522617	337.3893133	1.1783	1.24E-06	2.12E-05
3643931	CAALFM_C210170CA	6153.07826	4190.534681	0.55417	0.004151	0.02302
3643941	ACS1	20852.40718	7235.312054	1.5271	5.60E-07	1.02E-05
3643943	NA	298.649424	412.097962	-0.46453	0.006269	0.03173
3643945	RAP1	1256.125373	934.8495073	0.42617	0.001029	0.007647
3643946	CAALFM_C210070WA	695.2515339	988.4848681	-0.50768	0.006511	0.032678
3643954	KIP2	239.0929081	161.8469415	0.56294	0.006608	0.033115
3643955	CAALFM_C210300CA	385.0300963	180.0047394	1.0969	3.57E-09	9.30E-08
3643960	PUS7	1690.507802	2377.826653	-0.49219	0.000429	0.003682
3644010	CAALFM_C600850WA	2504.7285	1376.560701	0.86359	3.86E-13	1.54E-11
3644012	ILV5	14082.44448	7139.15786	0.98007	6.93E-20	4.46E-18
3644015	CAALFM_C601050WA	514.470418	704.6404724	-0.4538	0.00215	0.013813
3644017	CAN2	20539.09324	11144.04792	0.8821	1.32E-07	2.70E-06
3644018	CIP1	2329.081528	1309.508381	0.83074	5.57E-12	1.99E-10
3644027	PEX7	1762.266524	1147.604347	0.61881	6.23E-07	1.13E-05
3644028	NA	1877.807873	1216.867681	0.62588	2.73E-05	0.00034
3644032	CAALFM_C600930CA	3958.246221	2283.114613	0.79386	7.94E-06	0.000116
3644035	CAN1	13884.04124	7690.17822	0.85234	0.000612	0.004951
3644053	FAD2	8859.324541	12307.61638	-0.47428	8.80E-06	0.000126

3644059	CIC1	481.8984068	728.575653	-0.59635	0.00329	0.019152
3644061	DDR48	12613.54673	7274.783017	0.794	5.78E-12	2.05E-10
3644074	NA	1741.761092	615.50905	1.5007	3.73E-12	1.36E-10
3644077	ECM17	48675.01648	66622.70035	-0.45283	1.19E-05	0.000165
3644082	CAALFM_C206140CA	127.1202827	52.56459155	1.274	1.77E-05	0.000231
3644088	MET10	42704.89119	55400.6275	-0.3755	0.006201	0.03151
3644100	FAA2-1	11697.69472	3742.692043	1.6441	1.20E-35	1.43E-33
3644102	CAALFM_C209280CA	597.6386656	300.0652823	0.994	0.003281	0.019115
3644103	MED18	318.3353141	220.6975017	0.52848	0.005173	0.027348
3644104	NA	537.4155353	733.3779517	-0.44852	0.001764	0.011834
3644105	PES1	1550.116098	2147.426097	-0.47023	9.19E-05	0.000986
3644113	NA	793.3637484	538.8933465	0.55798	0.000225	0.002133
3644116	SPE2	1217.09555	905.2423913	0.42707	0.001098	0.008046
3644121	CAALFM_C500750CA	8819.651505	6218.48179	0.50416	3.14E-06	4.92E-05
3644123	FOL1	1058.712584	657.3218104	0.68764	4.63E-07	8.67E-06
3644126	CAALFM_C500800CA	3428.431876	4295.077066	-0.32514	0.003404	0.019699
3644128	NA	528.9809626	739.6340638	-0.4836	0.000822	0.006342
3644138	NA	2475.141822	3406.729308	-0.46088	5.77E-05	0.000646
3644150	CAALFM_CR08000CA	2527.369402	2065.338717	0.29126	0.01087	0.049191
3644404	NA	3548.766731	2609.595671	0.44349	7.71E-05	0.000841
3644405	MNN2	1664.180351	2083.102986	-0.32392	0.006903	0.034303
3644407	CAALFM_C110710CA	83.53706639	45.54865831	0.87501	0.009258	0.043194

3644419	NA	1835.731573	2556.768487	-0.47797	3.59E-05	0.000429
3644427	CAALFM_C110970WA	783.1396238	1138.73583	-0.54009	8.87E-05	0.000957
3644432	UBA4	1392.727362	2272.653484	-0.70646	4.49E-09	1.14E-07
3644434	NA	746.1732468	1143.734893	-0.61617	3.06E-06	4.81E-05
3644435	CAALFM_C110820CA	1678.604211	2098.232857	-0.32191	0.007234	0.035574
3644440	CDC48	53850.49092	41889.52876	0.36237	0.000533	0.004401
3644441	NA	6168.146653	4314.639449	0.5156	1.02E-05	0.000144
3644445	CAALFM_C110910CA	892.9842	1154.594792	-0.37068	0.004852	0.026004
3644449	CAALFM_C110880WA	721.344973	1133.516262	-0.65204	1.17E-06	2.02E-05
3644450	ADA2	855.1302176	641.9714343	0.41364	0.002945	0.017482
3644453	RME1	799.7872777	187.8059819	2.0904	1.50E-08	3.53E-07
3644462	NA	447.6772507	593.7848829	-0.40748	0.006808	0.033926
3644463	GAP4	10224.68952	16772.54553	-0.71404	1.35E-07	2.75E-06
3644470	CAALFM_C107360WA	2530.926744	1666.096405	0.60319	0.001994	0.013065
3644471	GPX3	610.9521085	364.6694844	0.74447	4.57E-05	0.000531
3644482	NA	2635.262758	3236.602395	-0.29653	0.009526	0.044224
3644484	PEX3	2194.085341	1483.113841	0.56499	2.29E-06	3.74E-05
3644530	NA	2541.965311	3261.575867	-0.35962	0.001596	0.010914
3644533	ADK1	7673.951959	5546.398871	0.46842	2.01E-05	0.000259
3644543	GIN4	753.0232783	554.3535383	0.44189	0.00741	0.03622
3644551	NA	4587.852747	3409.892538	0.42809	0.000145	0.001454
3644558	CAALFM_C601950CA	328.020711	450.0122854	-0.45618	0.005327	0.027954

3644560	NA	310.2895013	504.7369142	-0.70192	1.63E-05	0.000216
3644563	GPD2	13144.05424	29206.25719	-1.1519	1.04E-23	8.03E-22
3644566	CAALFM_C102270CA	2551.815836	603.1866004	2.0808	2.51E-59	6.87E-57
3644569	CAALFM_C102300WA	350.3334959	244.467522	0.51909	0.004804	0.025808
3644573	TIM21	402.9732369	559.2647789	-0.47285	0.002082	0.013511
3644576	CAALFM_C102370CA	388.6161203	249.154763	0.6413	0.000307	0.002785
3644579	HGT2	91.72806664	38.67327088	1.246	0.008455	0.040274
3644580	SHA3	9235.758534	15743.69397	-0.76947	0.005035	0.026739
3644592	ABP140	286.0619181	392.3941601	-0.45598	0.008058	0.038728
3644598	NA	4143.969991	6092.347769	-0.55598	3.44E-07	6.49E-06
3644682	CAALFM_C103790CA	3034.539885	3742.855456	-0.30266	0.007473	0.036448
3644702	ERB1	2546.833801	3615.579595	-0.50552	8.55E-06	0.000124
3644703	NA	71.16149136	29.15911672	1.2871	0.000935	0.007057
3644705	HHF1	1182.910816	884.3052808	0.41972	0.001456	0.010097
3644710	ACS2	18299.73484	12809.78719	0.51458	1.09E-06	1.89E-05
3644711	SSA2	43613.21927	10897.79855	2.0007	1.59E-07	3.21E-06
3644715	HTA2	2610.807583	1920.468114	0.44304	0.000585	0.004787
3644716	CAALFM_C104180WA	1206.414602	950.7067221	0.34365	0.008926	0.042033
3644718	CAALFM_C104200CA	1314.444086	1007.119648	0.38422	0.002339	0.014681
3644721	NA	598.6480572	1036.780361	-0.79233	7.47E-09	1.79E-07
3644760	DUR3	177.0780861	61.32067195	1.5299	8.63E-09	2.07E-07
3644761	CAALFM_C104640WA	2412.523669	1401.022966	0.78406	7.05E-11	2.19E-09

3644769	PIL1	23134.42209	15883.89219	0.54248	0.002249	0.014285
3644771	DUR1%2C2	17354.67945	7350.655953	1.2394	6.03E-21	4.16E-19
3644776	ERG3	3904.437241	3009.221027	0.37572	0.000783	0.006091
3644777	SYG1	1291.132289	808.219283	0.67582	0.000256	0.002361
3644778	IFE1	285.51495	126.8788383	1.1701	3.08E-08	6.91E-07
3644780	LPG20	3163.575786	1545.559753	1.0334	1.65E-17	9.09E-16
3644790	RPM2	18473.597	11798.17183	0.6469	0.010453	0.04752
3644840	CAALFM_CR06070WA	3961.547971	2048.168234	0.95173	1.03E-16	5.32E-15
3644841	NA	8684.357342	3400.331625	1.3527	1.84E-05	0.000238
3644846	CAALFM_CR06330CA	394.9790068	530.4166104	-0.42535	0.007448	0.036379
3644863	NA	6758.016541	5377.804857	0.32958	0.00223	0.01419
3644869	ZCF19	391.8051439	175.5406244	1.1583	1.72E-06	2.86E-05
3644870	NA	727.3810627	511.3031009	0.50853	0.000467	0.003955
3644911	NA	2429.397094	1228.244029	0.984	0.009022	0.042366
3644915	OPT2	835.6961405	339.5800808	1.2992	5.34E-18	3.08E-16
3644918	OPT3	4467.893631	1415.28058	1.6585	7.85E-11	2.42E-09
3644926	CAALFM_CR02420WA	173.4404794	255.6928405	-0.55997	0.008367	0.039976
3644933	ERG25	3227.888566	1605.305438	1.0077	8.99E-18	5.00E-16
3644934	IDP2	15095.50544	7362.798961	1.0358	6.13E-11	1.92E-09
3644941	NA	655.5893789	280.8691927	1.2229	3.47E-13	1.40E-11
3644946	PHO113	283.8519648	156.8794826	0.85548	2.34E-05	0.000295
3645002	RCH1	487.3242451	241.1558827	1.0149	3.99E-09	1.02E-07

3645003	NA	67.88578328	113.1834081	-0.73748	0.010644	0.048254
3645005	FRP1	1964.253968	2876.840656	-0.5505	2.18E-06	3.56E-05
3645006	NA	1143.719325	2174.955755	-0.92725	1.70E-14	7.63E-13
3645007	RBT5	2076.376061	4687.982434	-1.1749	3.06E-25	2.61E-23
3645022	MEP2	1890.665812	606.5790725	1.6401	3.50E-36	4.41E-34
3645026	PEX5	10160.23269	7664.119546	0.40674	0.000161	0.001598
3645027	CAR2	15689.87914	10768.48314	0.54302	2.92E-07	5.62E-06
3645099	IME2	20.98351668	4.939349806	2.0869	0.009997	0.046019
3645100	IFR2	3776.400671	2197.021601	0.78146	8.29E-12	2.88E-10
3645101	CAALFM_CR03270WA	286.0736187	180.0890669	0.66768	0.000733	0.005767
3645104	NA	156.3897246	263.6372499	-0.75341	0.000796	0.006172
3645105	NA	1458.576561	1965.93594	-0.43066	0.000403	0.003494
3645111	HGT18	416.1744543	236.0777345	0.81793	3.82E-06	5.92E-05
3645121	CAALFM_CR03130WA	523.6721302	363.5459915	0.52653	0.000922	0.006987
3645124	CAALFM_CR03110WA	729.8579898	954.185269	-0.38665	0.0041	0.022815
3645128	NA	687.5923411	523.1476946	0.39434	0.006753	0.033738
3645140	KTI12	654.2848321	841.0308438	-0.36224	0.010023	0.046077
3645199	NA	2134.053381	3191.357695	-0.58057	4.87E-07	9.07E-06
3645201	CAALFM_C207430CA	523.7505742	705.5083618	-0.42978	0.003013	0.017804
3645203	CAALFM_C207410WA	3230.552703	2201.874002	0.55305	2.39E-05	0.000299
3645204	1-Apr	32254.68034	24818.28861	0.3781	0.000293	0.002666
3645212	NA	1636.665359	1291.692261	0.3415	0.004752	0.025594

3645216	CAALFM_C207580WA	598.6012549	257.0965514	1.2193	1.12E-13	4.72E-12
3645217	RNR22	942.0716986	1305.555473	-0.47075	0.000222	0.002113
3645224	NOC4	2651.545554	3771.138821	-0.50817	0.000199	0.001922
3645261	KIP1	422.3171695	267.0876876	0.66101	0.000139	0.001406
3645271	CAALFM_CR07170WA	5832.137813	1555.635217	1.9065	1.33E-60	3.81E-58
3645278	GST3	1231.917731	767.3077632	0.68303	1.96E-07	3.88E-06
3645338	NA	15646.60934	10356.50168	0.59531	0.000445	0.003799
3645339	GAD1	16767.84069	23763.73699	-0.50306	0.000137	0.001406
3645348	PHO84	5770.811048	8721.104432	-0.59574	0.006212	0.031541
3645350	CAALFM_C403810WA	943.8226885	685.4971064	0.46137	0.000784	0.006093
3645360	CAALFM_C403710CA	1025.676829	587.748402	0.80331	4.79E-08	1.04E-06
3645361	CAALFM_C403700WA	1290.405032	788.4109076	0.7108	5.32E-08	1.15E-06
3645369	NA	902.9647521	596.5607007	0.598	4.65E-05	0.000539
3645375	ECM38	686.9703891	137.7123758	2.3186	1.32E-39	1.84E-37
3645413	CAALFM_C101750WA	3303.690136	2462.500077	0.42395	0.000229	0.002158
3645414	CTN1	65650.20135	15580.07733	2.0751	2.45E-18	1.46E-16
3645419	HSP30	13644.60784	7066.025946	0.94936	0.00427	0.023499
3645420	HGT1	1586.20047	5195.65668	-1.7117	9.66E-12	3.32E-10
3645432	CAALFM_C101840CA	2884.668615	2165.838624	0.41348	0.00035	0.003131
3645436	UGA2	4987.577638	3951.406153	0.33597	0.002161	0.013855
3645441	NA	187.2788095	304.9185573	-0.70324	0.000266	0.002442
3645448	NA	770.9092362	1186.472635	-0.62205	2.45E-06	3.95E-05

3645452	CAALFM_C204340CA	361.52153	89.4968416	2.0142	2.63E-21	1.88E-19
3645464	CAALFM_C204480WA	2482.708731	769.8911853	1.6892	1.12E-24	9.21E-23
3645465	ADH3	687.1823199	31.90944643	4.4286	9.97E-40	1.45E-37
3645466	LYS22	3374.936991	1868.689706	0.85283	2.37E-13	9.70E-12
3645577	NA	628.5944939	854.3327457	-0.44267	0.004511	0.024558
3645628	CAALFM_C303840CA	1634.919149	2123.28891	-0.37708	0.001619	0.010993
3645631	NA	768.879572	1023.334419	-0.41245	0.001851	0.012293
3645644	GTT11	1974.205018	1200.338467	0.71783	5.24E-09	1.31E-07
3645645	NA	4218.804691	3020.201227	0.48219	1.47E-05	0.000198
3645648	CAALFM_C303680WA	2037.172049	2540.295491	-0.31843	0.005899	0.030365
3645649	CAALFM_C303670WA	1052.656553	1495.289629	-0.50639	0.002429	0.015135
3645652	NA	699.5943169	932.520815	-0.41462	0.002544	0.015729
3645653	DAL9	3209.665431	1029.345412	1.6407	7.27E-42	1.11E-39
3645657	CAALFM_C303930WA	537.0618772	724.4795781	-0.43186	0.002664	0.01624
3645658	HTA1	2459.851117	1972.33085	0.31867	0.006623	0.033166
3645724	ALS2	1475.437466	4451.933682	-1.5933	1.20E-42	1.87E-40
3645732	MIS11	6233.224934	4275.245824	0.54397	5.44E-07	1.00E-05
3645739	SPS20	2654.663054	736.1827523	1.8504	7.44E-28	6.77E-26
3645741	NA	916.2244724	1292.837747	-0.49677	0.000109	0.001147
3645745	NMD5	3366.351631	4367.177679	-0.37552	0.000803	0.006216
3645747	NA	640.5717484	892.4728161	-0.47845	0.000517	0.004293
3645748	RLP24	1051.902434	1340.686989	-0.34997	0.006159	0.031417

3645749	CSP2	83.16346722	166.3936367	-1.0006	9.15E-05	0.000983
3645750	CAALFM_C400710WA	552.434486	365.0331562	0.59778	0.000133	0.001374
3645751	NA	686.1529872	468.4485539	0.55064	0.000219	0.002093
3645754	TOK1	2011.187876	2769.499586	-0.46158	7.85E-05	0.000855
3645755	CAALFM_C400660WA	441.6879634	627.4898211	-0.50656	0.000675	0.005384
3645758	MLH1	346.0436159	247.7791613	0.4819	0.007855	0.037942
3645759	CAALFM_C400780CA	2910.650648	2236.417847	0.38015	0.001001	0.007477
3645783	CAALFM_C208990CA	1869.358959	2338.318845	-0.32293	0.005843	0.030135
3645784	NA	1355.597177	2341.74292	-0.78865	5.10E-11	1.61E-09
3645790	ASN1	4458.590575	5602.255881	-0.32942	0.002503	0.015562
3645791	CAALFM_C209050CA	2786.360951	2169.539424	0.36099	0.001985	0.013023
3645797	CAALFM_C208920WA	3393.642491	4259.969291	-0.32801	0.002687	0.016324
3645873	CAALFM_C602290CA	1519.073585	1934.694124	-0.34891	0.004505	0.024544
3645881	CAALFM_C602350CA	1616.472383	2259.255023	-0.483	0.002116	0.013651
3645882	CAALFM_C602370CA	817.1813103	1083.840799	-0.40742	0.008707	0.04116
3645883	CAALFM_C602380WA	2536.946853	3435.516016	-0.43743	0.00011	0.001158
3645884	CAALFM_C602410WA	2723.089705	1689.862056	0.68834	4.71E-09	1.18E-07
3645886	CAALFM_C602140WA	434.3624876	319.5969345	0.44265	0.007667	0.037171
3645890	SWD3	327.1482663	223.9810319	0.54657	0.003163	0.01856
3645896	NA	834.4336156	614.6930105	0.44093	0.001695	0.011418
3645903	DBP7	3030.313289	4269.200606	-0.4945	1.15E-05	0.000159
3645905	GCN3	704.4862079	900.7808973	-0.3546	0.01022	0.046721

3645909	CAALFM_C701280CA	425.4476902	642.2815087	-0.59422	0.000102	0.001087
3645914	CAALFM_C701170CA	692.5626761	430.2531388	0.68676	5.21E-06	7.90E-05
3645951	NA	2788.945824	2191.516573	0.34779	0.002569	0.015798
3645955	CAALFM_C604510CA	346.5073596	492.6419591	-0.50765	0.001677	0.011317
3645970	CAALFM_C604530CA	1655.240832	2085.602081	-0.33342	0.004959	0.026428
3645986	CAALFM_C604480CA	973.9129924	1349.867328	-0.47095	0.000207	0.001986
3646024	FRE7	8267.540857	4140.866207	0.99753	5.73E-20	3.72E-18
3646025	NA	5701.371364	2899.050542	0.97573	8.74E-13	3.39E-11
3646026	HGT16	1626.465956	777.8320999	1.0642	2.41E-06	3.90E-05
3646028	CAALFM_CR07250CA	7610.753557	4370.686082	0.80018	3.33E-08	7.46E-07
3646030	CLG1	8877.133194	10907.53237	-0.29716	0.004629	0.025031
3646039	MRPL8	543.5032077	820.600572	-0.59439	2.76E-05	0.000343
3646083	NA	680.1355184	881.077969	-0.37345	0.005598	0.02912
3646084	NA	3223.977066	4315.673372	-0.42074	0.000164	0.001623
3646085	NA	543.6848171	724.7053251	-0.41462	0.004797	0.025793
3646086	CAALFM_C600390WA	1069.712088	1396.787219	-0.38489	0.002291	0.014462
3646130	CAALFM_C104560WA	1355.090091	986.9098812	0.4574	0.000316	0.002864
3646133	NA	1838.921917	2412.350252	-0.39158	0.000811	0.006275
3646140	CAALFM_C104460CA	6534.338764	1510.452852	2.1131	2.70E-38	3.61E-36
3646142	NA	540.1408151	258.640808	1.0624	2.26E-10	6.59E-09
3646146	ICL1	164151.576	72579.84908	1.1774	7.52E-18	4.22E-16
3646235	CAALFM_C703550CA	34.77850451	10.35568173	1.7478	0.00343	0.019824

3646236	NA	2997.665793	1775.843505	0.75534	0.000333	0.003006
3646282	NA	132.33073	36.59581376	1.8544	4.33E-09	1.10E-07
3646288	PEX11	8127.470669	3458.986446	1.2325	5.82E-29	5.62E-27
3646293	GLE2	1217.595716	1603.458097	-0.39715	0.00145	0.010075
3646345	NA	415.69901	582.7273457	-0.48728	0.001602	0.010926
3646347	NA	401.8173377	579.4719248	-0.5282	0.000633	0.005087
3646348	FUR1	636.4270557	839.1527719	-0.39894	0.004389	0.024035
3646354	ZCF13	3079.687702	1669.071608	0.88374	3.63E-14	1.57E-12
3646359	TEF4	91.52783641	41.84436437	1.1292	0.001885	0.012493
3646393	CRG1	2834.361433	1862.776146	0.60557	0.008262	0.039501
3646398	FDH1	15463.07048	27477.53847	-0.82943	3.06E-12	1.13E-10
3646407	CAALFM_C103870CA	447.2204272	201.3327571	1.1514	0.001317	0.00932
3646425	KTR4	1407.678249	1126.190254	0.32187	0.011074	0.049834
3646427	CSH1	1032.366512	607.2044636	0.7657	0.002167	0.013882
3646429	CAALFM_C104040CA	2043.831411	2582.784619	-0.33765	0.00403	0.02254
3646490	NA	578.3250544	788.3827984	-0.44702	0.001584	0.010845
3646495	NA	191.5218872	308.4550699	-0.68755	0.000338	0.003041
3646501	PCL2	60.06014173	26.66089581	1.1717	0.00565	0.029346
3646505	GCV2	22683.05647	12573.42517	0.85124	1.34E-15	6.57E-14
3646508	VCX1	1012.877392	680.1413612	0.57455	0.001947	0.012811
3646509	ERG1	2454.800199	1820.030541	0.43164	0.000458	0.003895
3646528	NA	23195.12495	16922.04758	0.45492	1.45E-05	0.000195

3646557	RTS1	3521.814688	2809.043367	0.32624	0.004188	0.023205
3646567	RTA2	1663.803292	1006.637424	0.72494	6.86E-09	1.68E-07
3646614	NMD3	2527.346001	3303.058307	-0.38618	0.000815	0.006294
3646616	CAALFM_CR06740WA	267.485125	177.2544097	0.59364	0.002961	0.017545
3646618	LSC2	7441.830368	4861.541286	0.61424	1.55E-08	3.63E-07
3646659	CAALFM_C107640CA	873.1353213	534.4002486	0.70828	0.003572	0.020394
3646661	BBC1	5419.564543	4220.82428	0.36065	0.00111	0.008065
3646674	PRY1	23.07168383	3.367857643	2.7762	0.000888	0.006752
3646680	CAALFM_CR05910WA	800.3906089	1081.625417	-0.43442	0.001051	0.007737
3646688	SDA1	1052.035921	1527.566242	-0.53805	1.85E-05	0.00024
3646692	DBP8	1619.544401	2140.604306	-0.40243	0.001408	0.009855
3646693	MTG2	715.7058837	963.1943319	-0.42846	0.001794	0.012002
3646695	CAALFM_CR05760CA	247.6341064	138.6373572	0.83689	8.58E-05	0.000929
3646696	CAALFM_CR05750WA	8726.373999	6539.05191	0.4163	0.000104	0.001104
3646711	CAALFM_C703470WA	279.180245	424.8095714	-0.60562	0.000427	0.00367
3646716	NA	180.2437085	112.2856622	0.68278	0.003806	0.021447
3646721	CPY1	19718.35591	15499.23724	0.34734	0.000855	0.006543
3646722	NA	289.5811987	149.9197677	0.94978	3.38E-06	5.28E-05
3646723	PRR2	382.7334585	258.0513893	0.56868	0.001196	0.008602
3646724	SHM1	6056.452383	4719.360427	0.35988	0.000896	0.006793
3646726	CAALFM_C703310WA	781.5255808	120.7044326	2.6948	2.04E-26	1.81E-24
3646807	CAALFM_C403410WA	1030.162159	1404.896789	-0.44759	0.00204	0.013315

3646814	NA	383.0472345	827.7579248	-1.1117	3.96E-14	1.70E-12
3646825	CAALFM_C112610WA	1012.707483	1337.935785	-0.40179	0.001609	0.010948
3646827	CAALFM_C112810WA	728.7922351	535.1583223	0.44554	0.002236	0.014214
3646831	CAALFM_C112700WA	364.8839226	504.4848054	-0.46737	0.00372	0.021057
3646835	CAALFM_C112660WA	942.5636238	732.3098034	0.36414	0.00813	0.038957
3646841	FRE10	1137.683236	4367.589857	-1.9407	1.81E-05	0.000235
3646842	COX11	1368.066357	1951.282177	-0.51228	2.13E-05	0.000271
3646847	CAALFM_C404270WA	442.1131453	306.5190323	0.52844	0.001615	0.010979
3646852	CAALFM_C404350WA	563.5193547	739.101737	-0.39131	0.006132	0.031354
3646855	CAALFM_C404250WA	2204.804851	1619.090438	0.44547	0.000165	0.001628
3646908	CAALFM_C206200CA	651.9331514	905.6332985	-0.4742	0.000612	0.004951
3646909	NA	1586.125486	1242.044907	0.35279	0.003859	0.021694
3646918	NA	1426.735267	1801.307065	-0.33633	0.005778	0.02987
3646920	MAK16	196.4571204	296.6948036	-0.59477	0.001869	0.0124
3646951	CAALFM_C405390WA	529.2210749	330.3189091	0.68001	6.06E-05	0.000674
3646962	OFD1	1268.600653	2083.437675	-0.71573	4.74E-09	1.19E-07
3646970	NA	183.0157907	269.1923802	-0.55667	0.004314	0.023681
3646989	CAALFM_C503690WA	1249.680582	940.0699487	0.41072	0.001444	0.010053
3647043	CAALFM_C501440CA	894.4668961	586.1259336	0.60982	2.19E-05	0.000278
3647044	PRC2	4106.743242	2058.694318	0.99627	1.72E-11	5.79E-10
3647045	NA	462.6996616	306.2370669	0.59543	0.000361	0.003205
3647050	CAALFM_C501470CA	290.658654	179.5268836	0.69513	0.000503	0.004202

3647052	SEF2	865.7045402	624.6560376	0.47081	0.000725	0.005726
3647061	GLR1	3898.124617	3078.659132	0.34048	0.002268	0.014372
3647069	CAALFM_C600800CA	2411.694567	1828.556506	0.39934	0.000706	0.005621
3647071	COX15	2931.841316	3821.097123	-0.38218	0.000589	0.004803
3647077	CTR1	7605.702957	4426.016007	0.78107	8.83E-13	3.41E-11
3647080	CAALFM_C600760WA	4121.689667	2726.515851	0.59618	0.005233	0.027619
3647083	FAT1	9433.527485	4709.752335	1.0021	2.72E-20	1.82E-18
3647085	SSF1	445.4638372	645.8426358	-0.53587	0.000378	0.003319
3647087	PCD1	1309.535714	771.0120572	0.76423	5.62E-09	1.39E-07
3647088	CAALFM_CR09700WA	1107.973451	609.1940985	0.86295	2.59E-07	5.03E-06
3647090	RTA4	149.0395273	80.93752535	0.88081	0.000928	0.007023
3647095	CAALFM_CR09590WA	1309.187655	720.1603773	0.86228	0.001723	0.011594
3647097	CAALFM_CR09580CA	5673.916833	3356.669927	0.75731	0.002076	0.013511
3647103	CAALFM_CR09520CA	1358.729838	1049.578046	0.37245	0.003805	0.021447
3647131	FET34	1338.246487	6183.815025	-2.2082	2.61E-29	2.60E-27
3647137	NA	3844.635013	1842.928303	1.0608	2.51E-09	6.60E-08
3647139	SLY41	1999.861575	2549.639243	-0.35039	0.002565	0.015798
3647152	FET99	1985.92486	560.4146336	1.8252	5.11E-44	8.37E-42
3647153	FET3	657.7174282	861.6851152	-0.38969	0.004787	0.02576
3647181	GLY1	6224.963229	11191.63514	-0.84628	2.20E-14	9.75E-13
3647183	CAALFM_C110460WA	2662.870034	3349.41951	-0.33093	0.003119	0.018332
3647184	CAALFM_C110470WA	2492.883412	3455.617715	-0.47113	3.31E-05	0.000401

3647194	GCA2	5060.709289	1768.643039	1.5167	1.30E-10	3.84E-09
3647225	CAALFM_C503850WA	856.813144	1194.135952	-0.47891	0.000236	0.002203
3647227	CAALFM_C503830CA	337.3173481	469.488593	-0.47698	0.003624	0.02062
3647233	CAALFM_C503770CA	1146.15769	2739.820374	-1.2573	4.45E-08	9.70E-07
3647240	CAALFM_C503710CA	1089.611229	695.8562829	0.64695	5.82E-05	0.000651
3647241	AAT21	17762.13687	8969.278869	0.98574	2.57E-20	1.74E-18
3647255	CAALFM_CR07670WA	776.8187593	1051.343681	-0.43658	0.000888	0.006752
3647298	NA	12759.64937	5977.461934	1.094	4.50E-17	2.40E-15
3647300	MNT4	455.3494644	333.9081453	0.44752	0.007738	0.037491
3647308	LYS5	221.1332866	146.6362375	0.59267	0.005941	0.030545
3647309	CAALFM_CR04880WA	1352.867117	937.5137621	0.52911	4.52E-05	0.000526
3647310	CAALFM_CR04870CA	398.7501003	157.1605742	1.3432	1.34E-12	5.08E-11
3647320	ARO10	2601.331476	929.1213535	1.4853	5.36E-05	0.000612
3647322	CAALFM_CR06840WA	1834.790245	2310.082088	-0.33233	0.005038	0.026739
3647326	HHT2	980.6563983	557.2452875	0.81544	3.82E-05	0.000453
3647327	HHF22	514.4071347	381.2531691	0.43216	0.006977	0.034568
3647343	YHB5	1407.366613	1846.853102	-0.39207	0.001027	0.007642
3647350	CAALFM_CR07750CA	2473.825575	3056.348167	-0.30507	0.009059	0.042507
3647353	YHB1	21426.20398	52949.81685	-1.3052	1.22E-22	8.99E-21
3647393	NA	2654.053622	1310.964815	1.0176	2.83E-17	1.52E-15
3647395	ZCF16	6631.600934	3937.384922	0.75212	1.01E-11	3.45E-10
3647396	CTN3	17327.51583	6243.470838	1.4726	7.16E-20	4.56E-18

3647397	AAP1	243.0642318	95.78455762	1.3435	2.30E-06	3.74E-05
3647399	CAALFM_C303980CA	177.2081128	106.3361298	0.73681	0.004239	0.023389
3647407	ALD6	10166.31262	3429.939766	1.5675	1.58E-45	2.96E-43
3647448	CAALFM_C503920CA	612.9232698	794.1909113	-0.37378	0.008504	0.040365
3647453	CAALFM_C307940WA	2340.866792	2935.222971	-0.32643	0.00443	0.024177
3647473	NA	448.6645614	1053.200633	-1.2311	3.26E-18	1.89E-16
3647474	IDP1	1206.509527	802.0527405	0.58907	7.23E-06	0.000106
3647478	NA	4216.63544	2283.848946	0.88463	7.16E-15	3.28E-13
3647480	NA	375.690117	157.5532288	1.2537	6.57E-11	2.05E-09
3647484	NA	1197.60297	1514.965322	-0.33914	0.006167	0.031432
3647519	NA	647.2917531	401.1792233	0.69017	8.63E-06	0.000124
3647523	CAALFM_C106270WA	496.2121812	653.6201375	-0.3975	0.008624	0.040876
3647531	RHD1	1342.583169	554.0706992	1.2769	3.27E-11	1.06E-09
3647532	CAALFM_C105010CA	2816.877757	3714.764488	-0.39917	0.000397	0.003457
3647535	RRP6	3312.477547	4665.663606	-0.49417	9.56E-06	0.000136
3647536	REI1	276.3366391	420.6845137	-0.60631	0.000357	0.003188
3647563	CAALFM_C602020CA	2325.554007	1511.568481	0.62153	2.14E-07	4.21E-06
3647591	RNR3	1549.147408	2093.65019	-0.43455	0.000243	0.002255
3647592	CAALFM_CR05560WA	850.927022	1091.057865	-0.35862	0.007533	0.036634
3647596	NA	3535.988556	4511.839285	-0.3516	0.005792	0.029917
3647598	NA	890.0937917	651.7394446	0.44966	0.001042	0.007686
3647609	NA	713.3377221	536.5620332	0.41084	0.004852	0.026004

3647647	RBP1	5118.110576	4165.986341	0.29695	0.006676	0.033403
3647664	BRG1	2265.01496	1455.865274	0.63764	8.95E-08	1.88E-06
3647672	GPI7	644.9496332	437.1012908	0.56122	0.006174	0.031446
3647677	RPA190	12341.03195	15711.91941	-0.3484	0.001001	0.007477
3647678	CAALFM_C110680CA	8878.091185	7341.115135	0.27425	0.009154	0.042925
3647682	CAALFM_C110620WA	4093.993567	5149.335095	-0.33088	0.002605	0.015958
3647684	PAN6	1605.351912	1030.352973	0.63975	0.002817	0.016891
3647685	CAALFM_CR02570CA	2497.613635	1197.894717	1.06	2.35E-18	1.42E-16
3647692	DBP2	1953.850374	2712.859165	-0.47349	0.000181	0.001756
3647697	OPT4	4637.299685	8601.016653	-0.89122	5.23E-16	2.66E-14
3647730	SUI1	12289.4479	8207.621199	0.58238	6.29E-08	1.34E-06
3647732	CKS1	654.752036	836.5992063	-0.35359	0.01099	0.049585
3647737	NA	2010.893541	1092.11815	0.88071	1.43E-05	0.000194
3647748	NA	9766.13355	6503.763971	0.58651	4.49E-08	9.76E-07
3647754	AYR1	1268.057145	866.9925052	0.54853	2.11E-05	0.000269
3647758	NA	292.0828471	420.2628763	-0.52491	0.002387	0.014953
3647761	ATO1	1243.206791	115.738721	3.4251	3.68E-08	8.13E-07
3647801	YAK1	3015.675677	1968.180305	0.61562	5.27E-05	0.000603
3647834	NA	2505.876159	2021.4134	0.30995	0.008483	0.040323
3647851	CAALFM_C403830WA	3696.46875	4786.639519	-0.37287	0.000729	0.005757
3647881	CAALFM_C500130CA	948.2308947	1388.961362	-0.5507	1.73E-05	0.000227
3647888	CDC11	475.8657772	343.7885923	0.46903	0.003516	0.02018

3647890	PGA25	903.4050947	540.6607291	0.74065	1.32E-07	2.70E-06
3647899	NA	339.3036701	138.946558	1.288	7.73E-10	2.13E-08
3647912	MPP10	402.6195788	617.0533066	-0.61598	0.002314	0.014579
3647918	EMC9	2128.178959	2840.641865	-0.4166	0.000372	0.003288
3647956	UTP20	6665.552977	8883.164889	-0.41435	0.00012	0.001249
3647960	CAALFM_C301180CA	1193.192124	516.2714335	1.2086	0.000474	0.003994
3647972	ALS4	5602.50453	20811.76255	-1.8933	5.58E-67	2.03E-64
3647994	CAALFM_C104370CA	1268.464526	1708.21924	-0.42941	0.000398	0.00346
3648021	AMO2	1585.378287	1186.057987	0.41865	0.000631	0.005083
3648025	NA	13803.47963	10411.50478	0.40685	0.000123	0.00127
3648026	GRE2	3118.766467	1986.897665	0.65046	2.17E-08	4.96E-07
3648060	CAALFM_C400050WA	1570.149546	1120.2732	0.48705	0.000257	0.002376
3648061	NA	219.7255744	337.2768766	-0.61823	0.000882	0.006733
3648065	NA	962.9126683	241.943813	1.9927	3.16E-06	4.95E-05
3648080	NA	1870.291227	1433.641258	0.38358	0.001427	0.009949
3648082	GUT1	4528.022337	2865.046165	0.66032	4.27E-07	8.02E-06
3648086	GDH3	16831.30593	7381.08173	1.1892	5.47E-21	3.82E-19
3648091	IPK1	1421.054976	1037.621889	0.45368	0.000411	0.003542
3648101	DBF2	1133.971965	881.8114283	0.36284	0.005338	0.027982
3648102	FRP3	6311.301241	1347.909297	2.2272	1.64E-05	0.000216
3648103	NA	1848.614234	1354.776821	0.44839	0.000264	0.002429
3648120	KCS1	854.5633085	1142.301325	-0.41868	0.001249	0.008944

3648123	MNT3	443.2140017	597.8800841	-0.43185	0.010516	0.047742
3648166	NA	4355.614651	3230.928712	0.43093	0.000133	0.001374
3648168	NA	291.4058523	400.0583515	-0.45718	0.007636	0.037088
3648179	SPB1	3349.859545	4479.647	-0.41928	0.000169	0.00166
3648194	NA	114.4460924	66.51213055	0.78298	0.007208	0.035498
3648195	CAALFM_CR07700WA	8412.024669	3209.938878	1.3899	7.95E-36	9.84E-34
3648197	CAALFM_CR09050CA	173.9358648	91.48909755	0.92688	0.00023	0.002162
3648207	CAALFM_CR04920WA	1092.970981	1596.154956	-0.54635	0.000245	0.002273
3648208	PEX14	5103.334697	2331.019199	1.1305	1.73E-11	5.80E-10
3648226	ALK8	821.931474	426.9133904	0.94507	2.38E-07	4.67E-06
3648238	NA	4976.289079	3575.987459	0.47673	1.44E-05	0.000195
3648267	CAALFM_C500560WA	2217.576106	1590.016523	0.47994	5.68E-05	0.000637
3648275	ERG10	2523.740036	1972.618058	0.35545	0.007772	0.037601
3648320	HGT14	2455.165557	1191.104531	1.0435	7.52E-18	4.22E-16
3648327	FOX2	87032.41222	29214.00772	1.5749	1.40E-49	3.29E-47
3648347	CAALFM_CR07160CA	3757.014716	2713.797252	0.46927	3.29E-05	0.000399
3648366	CAALFM_CR06570CA	674.5246105	1518.954202	-1.1711	3.19E-19	1.99E-17
3648367	NA	3246.205306	2465.145833	0.39708	0.000526	0.004353
3648378	NA	1125.442468	1405.853374	-0.32095	0.010503	0.047712
3648382	CAALFM_CR06930WA	771.1879104	548.1246616	0.49258	0.000623	0.005027
3648427	CTR2	1037.40227	1332.01611	-0.36064	0.004077	0.022724
6335362	RPL7	2763.91386	3663.595744	-0.40655	0.00035	0.003131

6335429	NA	3613.902513	2919.843612	0.30767	0.006138	0.031358
30514976	RPL6	1731.323716	1322.560795	0.38854	0.001275	0.009099
30514981	NA	1996.297632	2471.789358	-0.30823	0.007385	0.036155
30514985	NA	9960.659892	6863.342202	0.53733	4.90E-07	9.11E-06
30514999	RIB4	6362.917387	4736.328029	0.42592	0.000117	0.001222
30515002	NA	765.4421956	980.8189294	-0.35769	0.006874	0.03419
30515025	CAALFM_C107280CA	1.340968807	58.96212318	-5.4584	0.00095	0.007144
30515051	RPL2	172.2212968	102.1820892	0.75312	0.002255	0.014305
30515052	CAALFM_C111260CA	26447.33006	41236.94661	-0.64082	5.47E-07	1.01E-05
30515073	RPL14	258.9790285	407.748031	-0.65484	0.000175	0.00171
30515097	NA	167.4511921	90.84346045	0.88229	0.004255	0.023434
30515111	RPL39	12636.04541	8894.719503	0.50652	2.16E-06	3.54E-05
30515141	CAALFM_C206610CA	552.5831336	212.1671684	1.381	7.04E-16	3.52E-14
30515168	NA	128.9332357	72.79897289	0.82463	0.002817	0.016891
30515171	NA	754.7928891	1056.092382	-0.48458	0.005266	0.027747
30515173	NA	35.2188471	67.74718643	-0.94381	0.010185	0.046626
30515174	PRM9	884.6232909	260.9422648	1.7613	7.23E-30	7.53E-28
30515203	CAALFM_C304100WA	145.2883749	82.73301715	0.81238	0.007337	0.035973
30515218	CAALFM_C306390WA	888.7211812	687.4647479	0.37045	0.006817	0.03393
30515225	CAALFM_C306750WA	1180.622419	712.9485536	0.72768	4.36E-08	9.54E-07
30515244	NA	5662.558571	4571.005162	0.30894	0.005225	0.027598
30515247	CAALFM_C401990WA	1361.680069	1745.465059	-0.35822	0.003449	0.019899

30515264	CAALFM_C403950CA	5732.244287	3349.170022	0.7753	3.39E-12	1.24E-10
30515291	CAALFM_C500120WA	377.2746583	530.3595183	-0.49136	0.001692	0.011407
30515317	NA	1068.38628	447.4016279	1.2558	9.02E-19	5.58E-17
30515318	CAALFM_C504480CA	24178.88054	34474.25915	-0.51177	9.44E-07	1.66E-05
30515320	RPL43A	609.2527012	189.4345661	1.6853	2.39E-06	3.89E-05
30515345	NA	9536.503728	7791.81355	0.2915	0.006468	0.032513
30515347	CAALFM_C604250WA	6511.909793	2988.830223	1.1235	3.78E-24	3.02E-22
30515353	NA	931.2421029	1322.840139	-0.50641	7.20E-05	0.00079
30515358	NA	1461.465649	939.0878753	0.63808	6.18E-07	1.12E-05
30515359	HHO1	631.0198383	810.5540911	-0.36122	0.009816	0.045348
30515371	CAALFM_CR00890CA	21832.28005	16646.0889	0.39128	0.001507	0.01041
30515374	CAALFM_CR01300WA	2508.545895	3870.124328	-0.62553	0.000298	0.002705
30515431	NA	264.1777752	403.0599163	-0.60949	0.000454	0.003867
30515450	TAR1	5387.633924	1256.411462	2.1003	1.45E-21	1.06E-19
30515454	NA	9892.158395	5617.610626	0.81633	0.000827	0.006371
Novel00002	NA	53.50872558	14.95859524	1.8388	0.000132	0.001363
Novel00050		10.8378361	32.21777356	-1.5718	0.008258	0.039501
Novel00058	NA	99.02668114	48.43953384	1.0316	0.006703	0.033515
Novel00060		240.182064	66.31624009	1.8567	2.61E-14	1.14E-12
Novel00073	NA	49.91446115	21.94641932	1.1855	0.00985	0.045438
Novel00075	NA	4359.384424	3001.95648	0.53822	1.93E-06	3.19E-05
Novel00109	NA	1861.512056	1284.227455	0.53557	0.000589	0.004803

Novel00111	NA	126.4714694	192.2692233	-0.60432	0.00865	0.040972
Novel00114	NA	10830.88914	8722.473766	0.31234	0.003361	0.019512
Novel00128	NA	293.2972493	74.48290171	1.9774	6.86E-18	3.92E-16
Novel00150		25991.01472	21372.80782	0.28224	0.007506	0.036544
Novel00161	NA	918.5046293	602.5954343	0.6081	3.22E-05	0.000393
Novel00162		368.635075	212.3639325	0.79565	1.51E-05	0.000201
Novel00165	NA	45.39616933	17.00706952	1.4164	0.004027	0.02254
Novel00172	NA	520.5099678	905.3803161	-0.7986	1.27E-06	2.17E-05
Novel00178	NA	95.05881754	151.1258407	-0.66886	0.007638	0.037088
Novel00195	NA	62.03822324	20.71136345	1.5827	0.000287	0.002611
Novel00199	NA	64.66511803	8.727971234	2.8893	1.22E-08	2.90E-07
Novel00206	NA	109.7977738	186.739581	-0.76618	0.00102	0.007605
Novel00227	NA	33.38249288	11.5064101	1.5367	0.009221	0.043112
Novel00238	NA	22.69808466	62.72350913	-1.4664	0.00046	0.0039
Novel00244	NA	136.5621071	70.30075198	0.95795	0.00055	0.004527
Novel00252		423.945053	615.2270846	-0.53724	0.000411	0.003542
Novel00257		145.036562	248.1999251	-0.77509	0.000225	0.002133
Novel00272	NA	336.77038	627.1252757	-0.89699	7.45E-09	1.79E-07
Novel00274	NA	179.9918956	110.1528604	0.70842	0.00295	0.017497
Novel00296	NA	3879.402136	2901.23432	0.41917	0.000233	0.002186
Novel00299	NA	19.54416282	59.5805248	-1.6081	0.000185	0.001789
Novel00305	NA	493.3183128	68.84257016	2.8411	6.63E-14	2.82E-12

Novel00328	NA	385.8708994	276.9119163	0.47869	0.009243	0.043168
Novel00348	NA	81.20878689	169.5093856	-1.0617	0.000149	0.001488
Novel00352		499.0310658	300.1469887	0.73346	1.11E-05	0.000154
Novel00361		28.01861766	91.99506251	-1.7152	1.76E-06	2.91E-05
Novel00373	NA	32.41512325	8.840407892	1.8745	0.002624	0.016044
Novel00375	NA	132.4208746	198.9478466	-0.58726	0.008018	0.03859
Novel00378		737.5887064	464.7178982	0.66646	6.85E-06	0.000101
Novel00379	NA	730.8756219	419.7569113	0.80007	0.000164	0.001623
Novel00385	NA	27.2046759	67.15776766	-1.3037	0.00104	0.007686
Novel00386	NA	32.24999478	6.510841969	2.3084	0.000542	0.004476
Novel00393	NA	30.92072656	113.2677356	-1.8731	1.61E-08	3.75E-07
Novel00403	NA	97.7723968	35.16486742	1.4753	1.71E-05	0.000225
Novel00409	NA	113.3920383	175.3183721	-0.62866	0.008081	0.038809
Novel00426		399.449176	122.6975622	1.7029	1.32E-05	0.00018
Novel00429	NA	560.2739681	235.6279878	1.2496	0.001354	0.009542
Novel00443	NA	3646.727157	2375.53306	0.61835	5.56E-07	1.02E-05
Novel00448	NA	384.71154	234.112714	0.71657	5.61E-05	0.000632
Novel00458	NA	783.947465	287.3510518	1.4479	4.03E-06	6.20E-05
Novel00462	NA	22.75312749	69.01035146	-1.6007	8.70E-05	0.00094
Novel00466	NA	1809.375941	3395.475007	-0.90812	2.99E-15	1.42E-13
Novel00484	NA	893.6165323	580.7895608	0.62164	1.12E-05	0.000156
Novel00485	NA	676.3960665	389.6736869	0.7956	1.99E-07	3.93E-06

Novel00489	NA	97.13874422	308.5675065	-1.6675	1.26E-07	2.59E-06
Novel00494	NA	211.3963069	345.3029924	-0.70791	0.000111	0.001164
Novel00500		3386.331356	2298.213754	0.55921	1.06E-06	1.86E-05
Novel00501		56.87111813	107.5430765	-0.91915	0.002892	0.017249
Novel00502	NA	66.44642942	125.1949095	-0.91391	0.001106	0.008052
Novel00528	NA	451.6065525	324.4510832	0.47707	0.003691	0.020907
Novel00546	NA	999.0295012	610.5697002	0.71037	2.56E-07	4.99E-06
